# A trehalose biosynthetic enzyme doubles as an osmotic stress sensor to regulate bacterial morphogenesis

**DOI:** 10.1371/journal.pgen.1007062

**Published:** 2017-10-30

**Authors:** Ximing Chen, Lizhe An, Xiaochuan Fan, Furong Ju, Binglin Zhang, Haili Sun, Jianxi Xiao, Wei Hu, Tao Qu, Liping Guan, Shukun Tang, Tuo Chen, Guangxiu Liu, Paul Dyson

**Affiliations:** 1 Key Laboratory of Desert and Desertification, Northwest Institute of Eco-Environment and Resources, Chinese Academy of Sciences, Lanzhou, Gansu, China; 2 State Key Laboratory of Arid and Grassland Agroecology of Ministry of Education, Lanzhou University, Lanzhou, Gansu, China; 3 Key Laboratory of Extreme Environmental Microbial Resources and Engineering of Gansu Province, Lanzhou, Gansu, China; 4 School of Chemistry and Environmental Science, Lanzhou City University, Lanzhou, Gansu, China; 5 State Key Laboratory of Applied Organic Chemistry, College of Chemistry & Chemical Engineering, Lanzhou University, Lanzhou, Gansu, China; 6 Key Laboratory of Microbial Diversity in Southwest China, Ministry of Education and Laboratory for Conservation and Utilization of Bio-Resources, Yunnan Institute of Microbiology, Yunnan University, Kunming, China; 7 Institute of Life Science, Swansea University Medical School, Swansea, United Kingdom; Indiana University, UNITED STATES

## Abstract

The dissacharide trehalose is an important intracellular osmoprotectant and the OtsA/B pathway is the principal pathway for trehalose biosynthesis in a wide range of bacterial species. Scaffolding proteins and other cytoskeletal elements play an essential role in morphogenetic processes in bacteria. Here we describe how OtsA, in addition to its role in trehalose biosynthesis, functions as an osmotic stress sensor to regulate cell morphology in *Arthrobacter* strain A3. In response to osmotic stress, this and other *Arthrobacter* species undergo a transition from bacillary to myceloid growth. An *otsA* null mutant exhibits constitutive myceloid growth. Osmotic stress leads to a depletion of trehalose-6-phosphate, the product of the OtsA enzyme, and experimental depletion of this metabolite also leads to constitutive myceloid growth independent of OtsA function. *In vitro* analyses indicate that OtsA can self-assemble into protein networks, promoted by trehalose-6-phosphate, a property that is not shared by the equivalent enzyme from *E*. *coli*, despite the latter’s enzymatic activity when expressed in *Arthrobacter*. This, and the localization of the protein in non-stressed cells at the mid-cell and poles, indicates that OtsA from *Arthrobacter* likely functions as a cytoskeletal element regulating cell morphology. Recruiting a biosynthetic enzyme for this morphogenetic function represents an intriguing adaptation in bacteria that can survive in extreme environments.

## Author summary

For free living bacteria, little is known about how environmental cues are perceived and translated into changes in cell morphology. Here we describe how a biosynthetic enzyme involved in synthesis of an important intracellular osmoprotectant doubles as an osmotic stress sensing morphogenetic protein. This protein is involved in an adaptive response involving a growth transition in stress-tolerant bacteria, from growing as individual cells to forming non-separating branched cell aggregates. We demonstrate that the protein can self-assemble into large networks, consistent with its role as a morphogenetic protein, this assembly process being promoted by a metabolic product of the enzyme. Depletion of either this metabolite or the morphogenetic protein results in the inability of the bacteria to grow as individual cells in conditions of low osmolarity.

## Introduction

Trehalose (a-D-glucopyranosyl(1,1)-a-D-glucopyranoside) is a non-reducing disaccharide that functions as an important intracellular protectant against a variety of stress conditions including desiccation, dehydration, heat, cold, and oxidation [1]. At least four different pathways for trehalose biosynthesis have been reported, described as OtsA/B, TreY/Z, TreS, and TreT [2]. The OtsA/B pathway is the principal pathway for trehalose biosynthesis and is widely distributed in bacteria, fungi and plants (trehalose-6-phosphate synthase and trehalose-6-phosphate phosphatase in *Arabidopsis thaliana*). OtsA utilises UDP-glucose and glucose-6-phosphate to synthesize trehalose-6-phosphate (T6P) and subsequently OtsB converts T6P into Pi and trehalose. As a signaling molecule, T6P is important as an ‘energy checkpoint’ during development in eukaryotes. For example, in *Saccharomyces cerevisiae*, T6P controls the decision to proceed through cell division [3] and in plants it regulates flowering both in the leaf and in the shoot apical meristem [4].

Osmotic stress can inhibit the growth rate and affect the morphology of bacteria belonging to several genera, including *Arthrobacter* species, *Rhodococcus* species [5] and *Aeromonas hydrophila* [6]. However, the relationship between osmotic stress, trehalose biosynthesis and the regulation of cell division and morphogenesis is unclear. Cell division and dynamic reorganisation of cell morphology depends on the internal cytoskeleton or scaffolding elements. In most bacteria, the tubulin homolog FtsZ is critical for driving binary fission [7]. The FtsZ protein assembles into protofilaments that are bundled together to form the Z-ring at the site of cell division, usually at the mid-cell. Other components of the division machinery are then recruited to form a multi-protein divisome complex responsible for mid-cell peptidoglycan synthesis. Contraction of the Z-ring also drives constriction of the cell envelope to form the septum [8]. Other cytoskeletal elements that have a role in determining cell morphology in typical rod-shaped eubacteria include MreB, an actin-like ATPase cytoskeletal proteins, that *in vitro* can polymerize into filaments in the presence of ATP or GTP [9, 10] and guide lateral wall peptidoglycan synthesis [11, 12].

The actinobacteria include species of contrasting morphologies, including coccoid-shaped *Rhodococci*, rod-shaped *Mycobacteria* and *Corynebacteria*, filamentous spore-forming *Streptomyces* and pleomorphic *Arthrobacter*. Actinobacteria studied thus far grow by apical extension, with new peptidoglycan synthesized and added at the cell poles. This contrasts with other eubacteria that insert new peptidogylcan in their lateral walls, with the poles being inert [13–15]. Apical growth in actinobacteria is independent of MreB. In fact, the genomes of actinobacteria that adopt bacillary-type growth lack *mreB* homologs, and filamentous *Streptomyces* use an MreB protein only during sporulation [16–18]. Apical growth is guided by the protein DivIVA [13–15]; DivIVA assembles to form an internal cytoskeletal element at the cell poles that appears to function to recruit proteins for apical cell-wall synthesis.

Actinobacterial *Arthrobacter* species typically inhabit soil ecosytems and are fascinating for their pleomorphism. During exponential growth, rod-shape cells elongate and undergo cell division at the midcell region; the two daughter cells remain joined forming a V shape and subsequently separate by snapping apart [19]. A reversible transition from rod-shaped cells to non-separating multi-cellular, branching myceloids is induced in some species by osmotic stress and this is documented to be an adaptive response to promote bacterial survival through altered metabolism and increased resistance to environmental stress [20, 21]. Indeed, *Arthrobacter* typically exhibit high resistance to, among other stresses, cold, heat and dessication [22, 23]. Stress resistance is likely related to their pleomorphic behaviour, making them an interesting model for analysis of environmentally triggered developmental switches although, to date, there has been a paucity of molecular characterization of these bacteria. *Arthrobacter* sp. strain A3 (hereafter referred to as *Arthrobacter* A3), a psychrotrophic bacterium, was isolated from the alpine permafrost of the Tianshan Mountains in China [24]. It has an optimal growth temperature of 20 ^0^C, but can survive and grow at near-freezing temperatures as low as -4 ^0^C. Its stress tolerance is in part due to synthesis of trehalose catalyzed by OtsA/B [25**]**.

As in *Escherichia coli* [26], the *otsAB* genes in *Arthrobacter* A3 are arranged as an operon [25]. This organisation allows for efficient co-regulation of both genes [27]. OtsA of *E*. *coli* contains an N-terminal loop, located between Arg9 to Gly22, based on the crystal structure. This N-loop is located in the catalytic centre of the OtsA enzyme and interacts with the phosphate moiety of glucose-6-phosphate and the distal phosphate of UDP-glucose, respectively [28]. Furthermore, both ends of the amino acid sequence of the N-loop are conserved in many microorganisms. During the catalytic reaction, the N-loop undergoes significant conformational changes [29], suggesting that the N-loop is directly related to the catalytic efficiency of OtsA. The enzymatic activity of OtsA of *Arthrobacter* A3 at low temperatures is due to a very flexible N-loop containing the active site [25], a key feature that distinguishes the protein from its *E*. *coli* counterpart.

Here we demonstrate that depletion of OtsA or T6P results in constitutive myceloid growth. Further analyses indicate that OtsA doubles as a novel self-assembling morphogenetic protein. OtsA, acting as an osmotic stress sensor together with T6P, mediates the switch to myceloid growth during osmotic stress. Recruiting a biosynthetic enzyme for this morphogenetic function represents an intriguing adaptation in bacteria that can survive in extreme environments.

## Results

### An *otsA* mutant of *Arthrobacter* A3 exhibits a constitutive myceloid morphology

**Fig 1:**
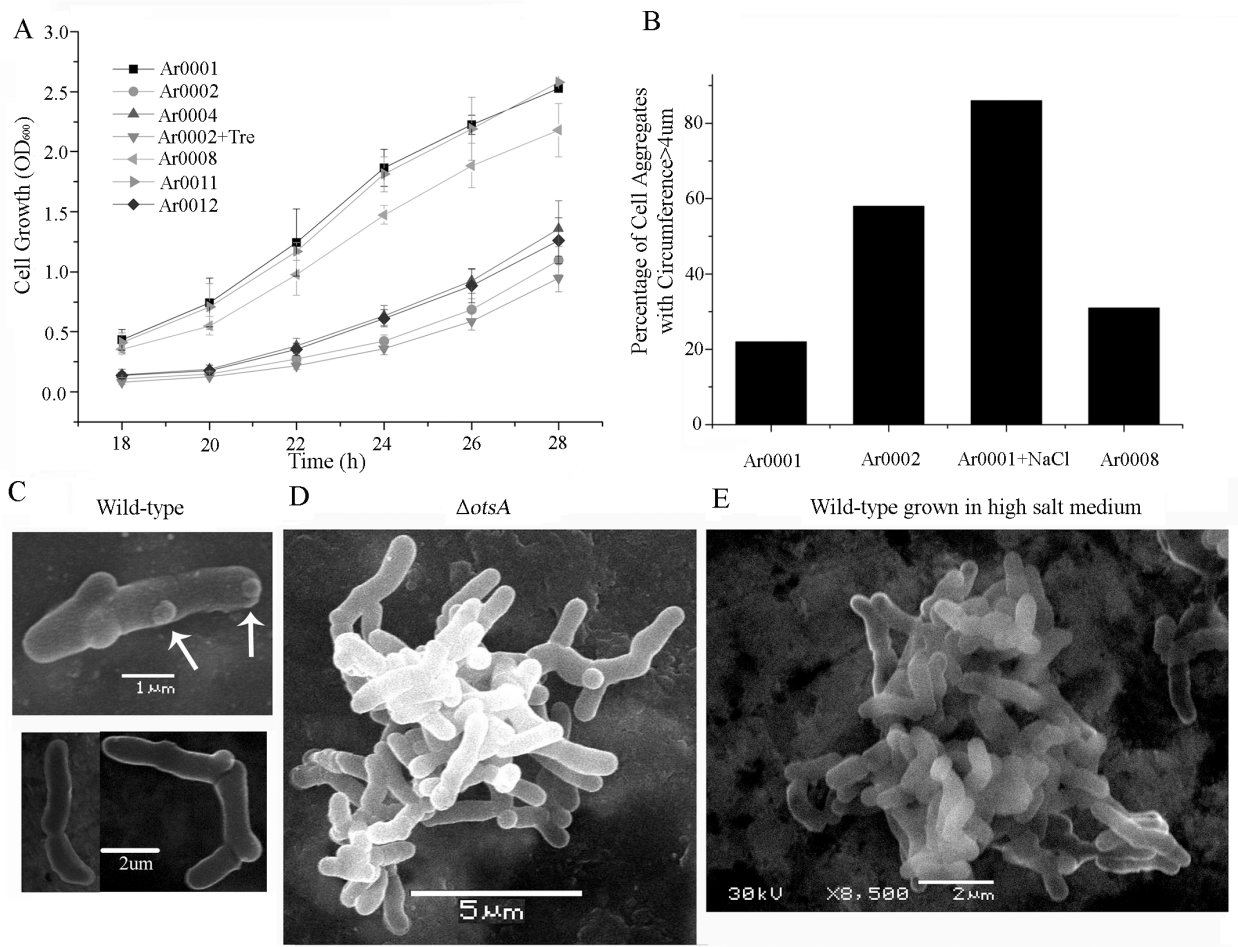
Apparent growth rate reflects bacillary growth or myceloid formation. (A) Growth curves of wild-type (Ar0001), Δ*otsA* (Ar0002), wild-type + up-*otsA* (Ar0004), wild-type + *treF*_*Ec*_ (Ar0008), wild-type + up-*otsA*_R36A_ (Ar0011), wild-type + *treC*_*Ec*_ (Ar0012) grown in LB and Δ*otsA* (Ar0002) grown in LB supplemented with 4mM trehalose. (B) The percentage of cell aggregates with circumference >4 um in early log-phase cultures of wild-type (Ar0001), Δ*otsA* (Ar0002), wild-type + *treF*_*Ec*_ (Ar0008) grown in minimal medium and wild-type (Ar0001) grown in minimal medium amended to a final concentration of 0.57 M NaCl. (C) SEM of wild-type (Ar0001) early log-phase cells. (D) SEM of an early log-phase myceloid of Δ*otsA* (Ar0002) grown in LB. (E) SEM of an early log-phase myceloid of wild-type (Ar0001) grown in LB amended to a final concentration of 0.57 M NaCl.

An *otsA* deletion mutant (Ar0002) has significantly reduced intracellular trehalose levels compared to the wild-type strain, Ar0001 (S1A Fig) [24]. We also observed that the mutant exhibits an apparent markedly slower growth rate in low osmolarity medium as determined by optical density (OD_600_; Fig 1A). Whereas the doubling time for the wild-type was 2.5 h, for the mutant it was 3.3 h, approximating the 3.2 h doubling time of the wild-type grown in salt-amended Luria broth (LB). When early log-phase cells were examined by phase-contrast microscopy, we observed extensive aggregate formation by the mutant, similar to previously reported myceloids formed by other *Arthrobacter* species after osmotic stress [30]. Hence the mutant fails to grow by snap division, but instead adopts non-separating myceloid growth, characteristic of the morphological switch of the wild-type when subjected to salt stress. Consequently, OD_600_ measurements do not necessarily reflect slower growth of the mutant or wild-type subject to salt stress, but simply the growth of cell aggregates. We subsequently employed OD_600_ measurements as a proxy for measuring the formation of myceloids, verifying the presence of cell aggregates in early log-phase cultures by phase-contrast microscopy. Quantification and analysis of aggregate dimensions of early log-phase cells of the *otsA* mutant grown in chemically-defined minimal medium revealed 58% of colony-forming units existing as myceloids with a maximum dimension between extremities of the aggregates of >4 μm (as viewed in two dimensions under the microscope), with the average value being 4.34 μm (n = 385; Fig 1B). In contrast, the proportion of wild-type cell aggregates of greater than 4 μm maximum dimension formed was 21% of the total, with 79% of colony forming units being single cells or small multiples of 2 to 4 cells still joined prior to snap division (Fig 1B). The aggregates of the mutant resembled those formed after 16 h growth by osmotically stressed cultures of the wild-type which have an average maximum dimension of 6.4 μm (n = 200), with over 80% of aggregates being > 4 μm in maximum dimension (Fig 1B). Similar proportions of cell aggregates were observed during growth in LB medium with or without addition of salt (S2 Fig). Cells of the wild-type, salt-induced wild-type myceloids and constitutive myceloids of the mutant were examined by scanning electron microscopy (Fig 1C–1E). Although many single or small chains of non-separated cells of the wild-type grown in LB appeared normal (Fig 1C, bottom left), we also detected a small proportion of cells in chains with branches emerging from their lateral walls (e.g. Fig 1C, top panel, arrowed). The much larger constitutive or salt-induced myceloids consisted of networks of extensively branched chains of cells (Fig 1D and 1E). Genetic complementation of the mutant with a single copy of *otsA* under control of its native promoter sequence restored both the normal growth rate and the largely bacillary morphology of log-phase bacterial cells grown in LB (S3 Fig), whereas complementation with the flanking genes, *otsB* or *dsbA*, or *E*. *coli otsA* (*otsA*_*Ec*_) did not restore bacillary growth (S3 Fig), although the latter gene was biochemically functional (see below).

### Myceloid formation is not dependent on intracellular trehalose concentration

To examine a possible link between intracellular trehalose and growth morphology, cultures of the Δ*otsA* mutant were subjected to a biochemical complementation test. Addition of between 0.5 mM and 4.0 mM trehalose, which is effectively taken up by the bacteria ([24], S1A Fig) had no effect on growth rate (Fig 1A) or myceloid formation. In addition, when a trehalase enzyme encoded by *treF* was over-expressed in the wild-type (strain Ar0008), resulting in a more than 5-fold reduction in intracellular trehalose to less than that detected in the Δ*otsA* mutant (S1 Fig), there was only a modest reduction in growth rate (Fig 1A). The growth rate was reflected in a low proportion (24%) of cell aggregates with maximum dimension > 4 μm (Fig 1B). We also determined that trehalose biosynthesis in the Δ*otsA* mutant was restored due to complementation by *otsA*_*Ec*_ (S1 Fig). Consequently, we concluded that the constitutive myceloid formation of the Δ*otsA* mutant did not reflect a reduction of intracellular trehalose. We also observed that the Δ*otsA* mutant is osmotic stress- sensitive and whereas this phenotype could be rescued by genetic complementation, it could not be by biochemical complementation with trehalose (S4 Fig).

### OtsA overexpression disrupts normal cytokinesis

**Fig 2:**
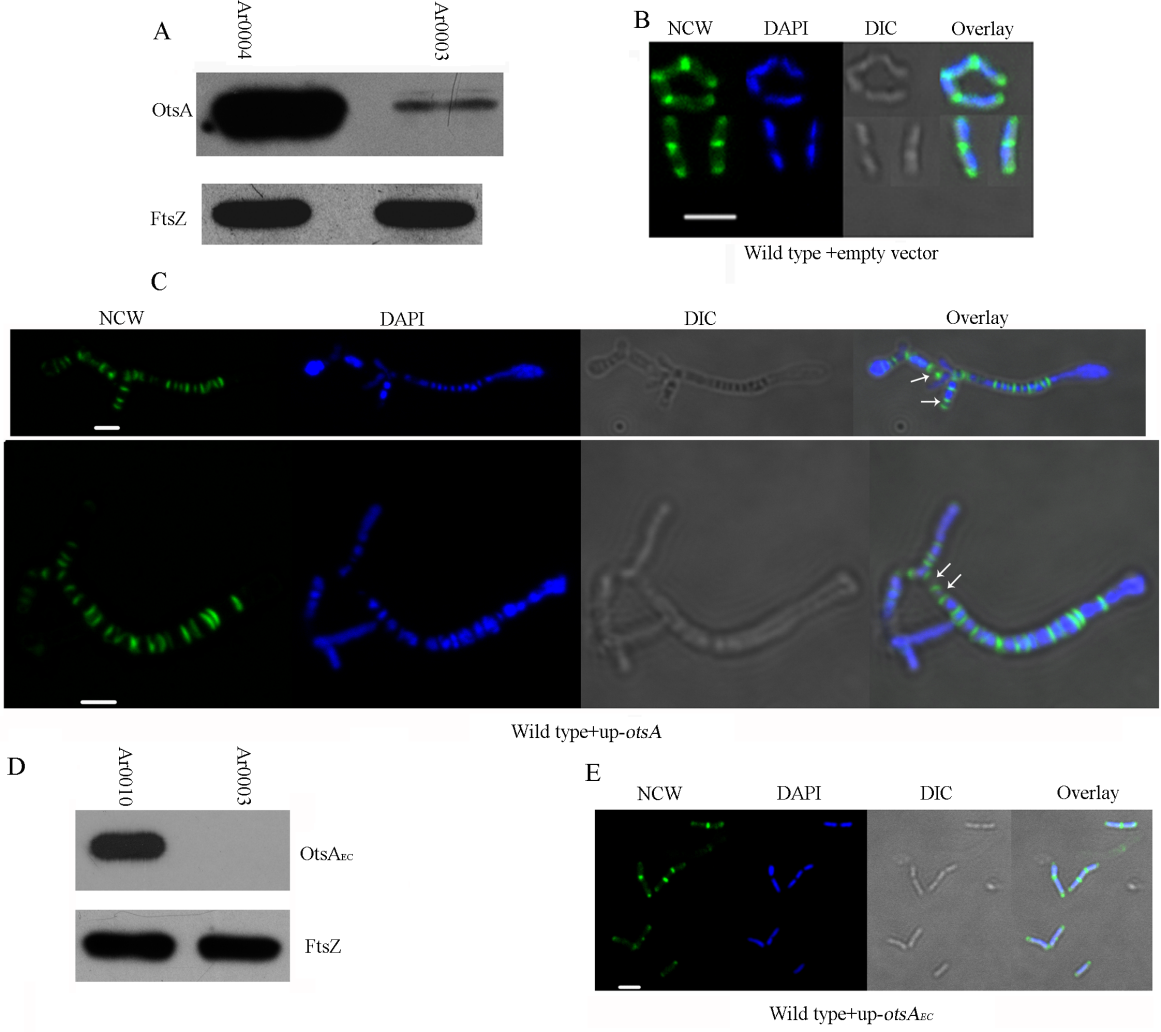
OtsA overexpression impacts on cell division. (A) Western blot to measure relative expression of OtsA and FtsZ in strains Ar0004 (wild-type + up-*otsA*) and Ar0003 (wild-type + empty vector). For each panel, the proteins were detected using antibodies against OtsA and FtsZ respectively. The strains were grown to early log phase in LB medium before preparing protein extracts. (B) Fluorescence microscopy images of fluo-vancomycin staining of nascent cell walls (NCW, green), DAPI staining of nucleoids (blue), DIC and the corresponding merged images of Ar0003 (wild-type with empty vector) cells grown to early log-phase in LB. (C) NCW, DAPI, DIC and merged images of representative Ar0004 (wild-type + up-*otsA*) cells. White arrows indicate cell compartments not staining with DAPI. (D) Western blot to measure relative expression of OtsA_*Ec*_ and FtsZ in strains Ar0010 (wild-type + up-*otsA*) and Ar0003 (wild-type + empty vector). For each panel, the proteins were detected using antibodies against the His-tag of OtsA_*Ec*_ and against FtsZ respectively. The strains were grown to early log phase in LB medium before preparing protein extracts. (E) NCW, DAPI, DIC and merged images of representative Ar0010 (wild-type + up-*otsA*_*Ec*_) cells. Scale-bars in (B), (C) and (E) represent 2 μm.

We constructed a strain, wild-type + up-*otsA*, containing the gene fused with a strong promoter on a multi-copy plasmid. Overexpression was verified by western blot, indicating an approximate 10-fold greater intracellular abundance of the protein relative to FtsZ (Fig 2A). We used a fluorescent derivative of vancomycin (fluo-vancomycin) that binds to nascent peptidoglycan to establish firstly if, as in other studied actinobacteria, growth is at the cell poles, and secondly to visualise how overexpression of OtsA affects cell wall biosynthesis. The antibiotic bound to nascent peptidoglycan at the poles, confirming apical growth, and, with more intense fluorescence, at the newly forming septum in the midcell region of rod-shaped wild-type cells (strain Ar0003) growing in LB (Fig 2B). In cells with evidence of septum formation, the distance between the stained poles and midcell was on average 0.7 μm. There was evidence for some ‘mini-chains’ of cells, for example the 4 joined cells in the top panel of Fig 2B, due to inefficient snap division. The result of OtsA over-expression (wild-type + up-*otsA*; strain Ar0004) was a pattern of peptidoglycan synthesis consistent with the formation of multiple septa in very long, enlarged cells with bulbous poles and limited branching (Fig 2C). Staining these cells with both DAPI and fluo-vancomycin revealed that many of the newly-formed compartments possessed less intensely staining nucleoids, with a small proportion lacking detectable DNA (arrowed in the overlay image, Fig 2C). This indicates that overexpression of OtsA affects the coordination of DNA synthesis and chromosome segregation with septum formation. The doubling time of this OtsA overexpression strain was 3.1 h (Fig 1A), indicating that these observed abnormalities in cell division can retard growth.

These results, indicating that OtsA function has a role on growth and division, prompted us to test the effects of overexpression of OtsA_*Ec*_. Over-expression of C-terminal His-tagged OtsA_*Ec*_, verified by western blotting, had no effect on septum formation or the rod-shaped morphology of the strain (wild-type + up-*otsA*_*Ec*_; Ar0010) grown in LB (Fig 2D and 2E). We tested the activity of the overexpressed OtsA_*Ec*_ in *Arthrobacter* A3 by analysis of the trehalose concentration in Ar0010; this strain had almost 5 times greater intracellular trehalose than the wild type strain (S1C Fig). Moreover, whereas the strain overexpressing OtsA was sensitive to osmotic stress, the strain overexpressing OtsA_*Ec*_ was not (S4 Fig). Consequently, we inferred that specific features of the *Arthrobacter* protein confer its function as a morphogenetic determinant.

### Depletion of trehalose-6-phosphate also leads to constitutive myceloid growth

**Fig 3:**
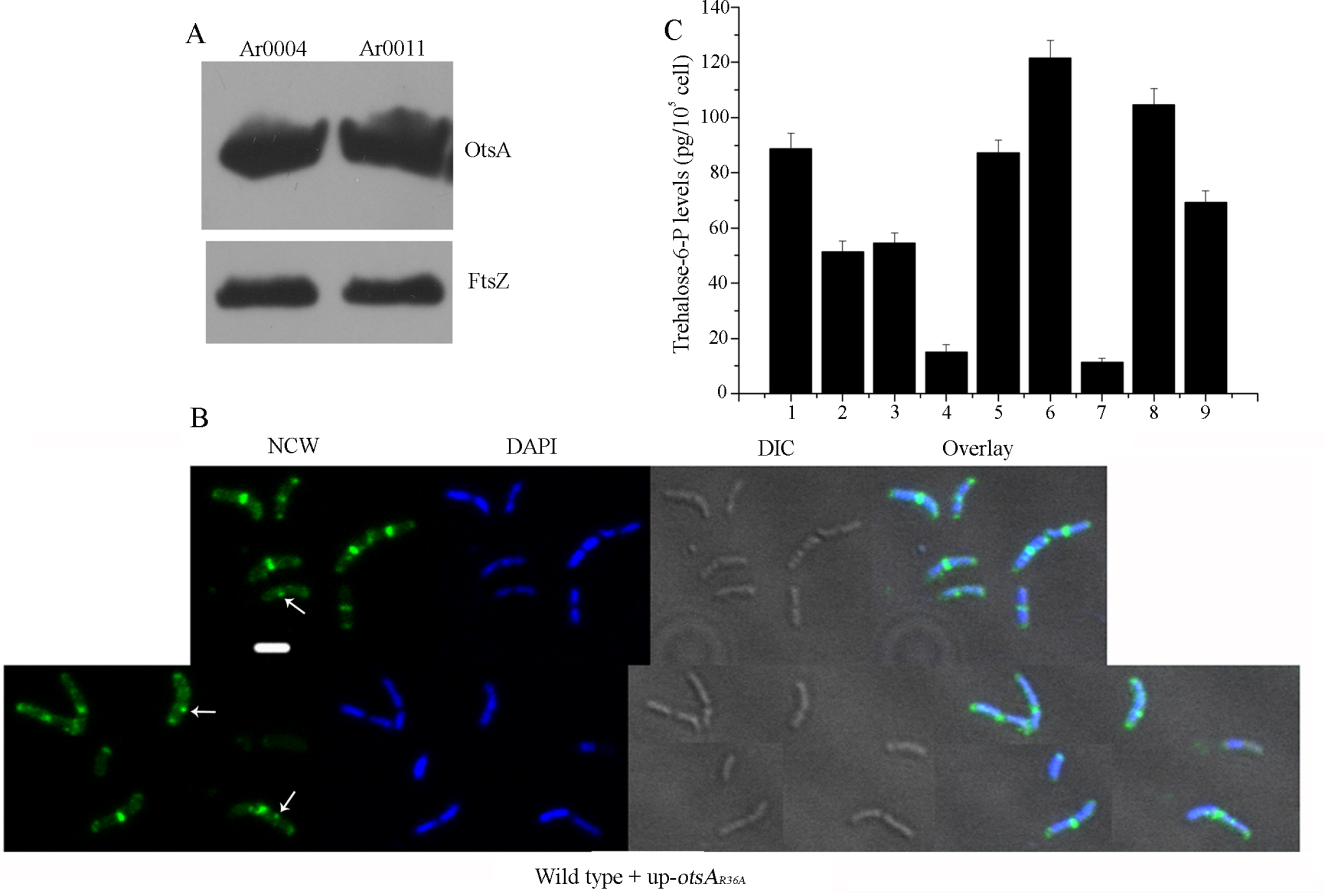
Enzymatically active OtsA and its product, trehalose-6-phosphate, impact on morphogenesis. (A) Western blot to measure relative expression of OtsA and FtsZ in strain Ar0004 (wild-type + up-*otsA*) and Ar0011 (wild-type + up-*otsA*_R36A_). For each panel, the proteins were detected using antibodies against OtsA and FtsZ respectively. The strains were grown to early log phase in LB medium before preparing protein extracts. (B) NCW, DAPI, DIC and merged images of representative Ar0011 (wild-type + up-*otsA*_R36A_) cells. Scale-bar represents 2 μm. (C) Intracellular T6P concentrations were determined in early log-phase cells grown in LB medium unless stated otherwise: 1. strain Ar0003 (wild-type with empty vector; 100%), 2. strain Ar0003 sampled after additional 3 h growth in LB medium amended to a final concentration of 0.57 M NaCl, 3. strain Ar0003 sampled after growth in LB medium amended to a final concentration of 0.57 M NaCl, 4. strain Ar0012 (wild-type with *treC*_*Ec*_), 5. Ar0011 (wild-type with up-*otsA*_R36A_), 6. Ar0004 (wild-type with up-*otsA*), 7. Ar0002 (Δ*otsA*), 8. Ar0010 (wild-type with up-*otsA*_*Ec*_), 9. Ar0112 (Δ*otsA* with *otsA*_*Ec*_).

To investigate the relationship between OtsA enzyme activity and its role as a morphogenetic protein and effector of the osmotic stress response, an amino acid substitution was introduced in the active site, as determined by crystallography of the corresponding *E*. *coli* enzyme [29], replacing the conserved arginine residue (R36) with alanine (the arginine residue of the *E*. *coli* protein is involved in glucose-6-phosphate binding). The mutant protein, OtsA_R36A_, was overexpressed in strain Ar0011 (wild-type + up-*otsA*_*R36A*_) at similar levels to OtsA in strain Ar0004 (Fig 3A). However, the Ar0011 strain had a much shorter doubling time compared to strain Ar0004, and similar to that of the wild-type (Fig 1A). Staining with fluo-vancomycin revealed single septa located at the midcell of dividing bacillary-form cells (Fig 3B), together with evidence of occasional foci located in the lateral walls (indicated by white arrows in the NCW image). The mutant protein was also expressed under control of the native promoter in the Δ*otsA* mutant (Δ*otsA* + *otsA*_*R36A*_; strain Ar0115). It could not restore normal bacillary growth to the mutant (S1 Fig). Moreover, when the mutant protein was overexpressed this did not affect bacillary growth or sensitivity to osmotic stress (S4 Fig).

**Fig 4:**
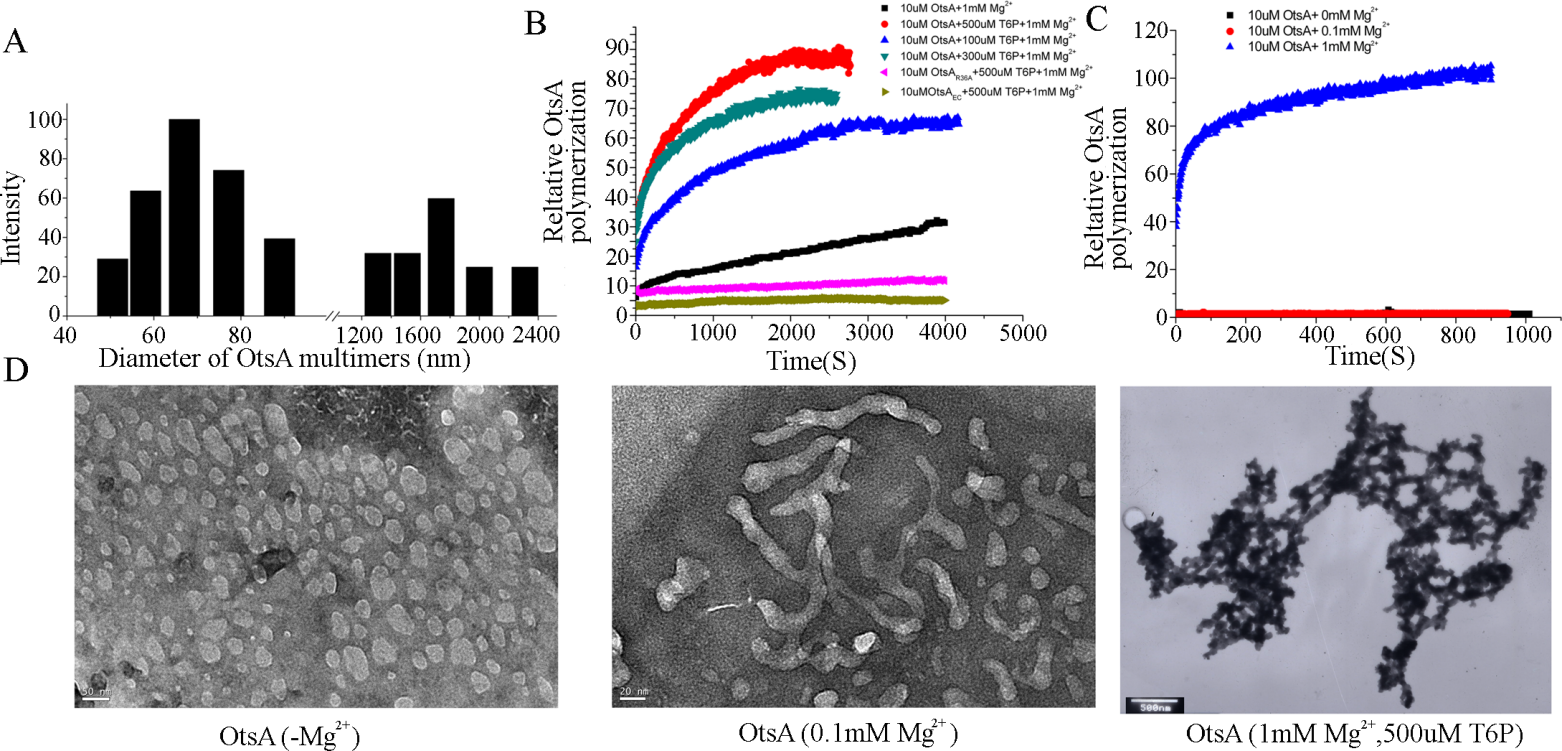
*In vitro* self-assembly of OtsA is promoted by T6P. (A) The diameters and quantities of non-denatured purified OtsA structures were determined by dynamic light scattering (DLS), revealing two populations of protein assemblies. (B) Self-assembly of OtsA, OtsA_R36A_ and OtsA_*Ec*_ in 1M urea and 1mM MgCl_2_ with different concentrations of T6P was measured using dynamic light scattering. (C) Self-assembly of OtsA with different concentrations of MgCl_2_ was measured using dynamic light scattering. (D) Transmission electron microscopy of small OtsA assemblies obtained after dialysis in the absence of Mg^2+^ ions (negatively stained, scale bar = 50 nm); partially polymerized OtsA assemblies obtained after incubation with 0.1 mM Mg^2+^ (negatively stained, scale-bar = 20 nm); and fully polymerized OtsA networks after incubation with 1 mM Mg^2+^ and 500 μM T6P (positively stained, scale bar = 500 nm).

We hypothesised that the loss of a morphogenetic function of OtsA_R36A_ *in vivo* could reflect an inability to synthesise a threshold concentration of trehalose-6-phosphate (T6P) that may be necessary for the morphogenetic function of OtsA. To examine this we monitored intracellular levels of T6P in wild-type cells before and after salt stress. Based on a cell volume of 10^−15^ l, the T6P concentration can be estimated as ranging from approximately 2 mM in the wild-type (no salt stress) to 0.2 mM in the *otsA* mutant. An approximate 40% decrease in the intracellular concentration of the metabolite was noted in wild-type cells 3 h after salt stress (Fig 3C, bars 2 and 3, compared to bar 1), coincident with the time when we noted changes in cell morphology (see below). To examine this further, we overexpressed the *E*. *coli treC* gene [31] encoding T6P hydrolase in the wild-type (wild-type + *treC*_*EC*_; strain Ar0012). The recombinant strain grew slowly and formed constitutive myceloids in the absence of salt-stress (Fig 1A and see below, Fig 4C). These cell aggregates had an average maximum dimension between extremities of 5.4 μm (n = 361), similar to those formed by the Δ*otsA* null mutant strain. Measurements of intracellular T6P revealed a significant depletion (17% of the level in the wild-type) of this metabolite in Ar0012 compared with the wild-type (Fig 3C, bar 4), indicative of functional activity of TreC_*Ec*_ in *Arthrobacter*. The reduced level of T6P in Ar0012 was comparable to the amount detectable in the Δ*otsA* mutant strain (Fig 3C, bar 7). We also compared the levels of this metabolite in the strains overexpressing OtsA and OtsA_R36A_. Whereas the former strain, Ar0004, contained 137% of the amount in the wild-type (bar 6), reflecting increased synthesis due to amplification of the enzyme, the latter had levels similar to wild-type, reflecting expression of the single-copy wild-type gene in this strain and an absence of metabolic activity due to the active site mutation in the overexpressed enzyme (Fig 3C bar 5; relative to OtsA, purified OtsA_R36A_ exhibited 7.53% +/- 0.22 enzyme activity). Quantification of T6P in strain Ar0010 overexpressing OtsA_Ec_ revealed 118% of the levels found in the wild-type (Fig 3C, bar 8), implying that an increase in the metabolite in the absence of increased amounts of OtsA protein is insufficient to promote any change in cell morphology. Moreover, expression of *otsA*_*Ec*_ in the Δ*otsA* mutant strain restored T6P synthesis (bar 9) but, as described above, did not change the constitutive myceloid phenotype of the mutant.

### Septum formation is reduced during myceloid growth

We used fluo-vancomycin to stain myceloids. Due to the extensive three-dimensional structure of myceloids of the Δ*otsA* mutant (strain Ar0002), fluorescence microscopy was more challenging and better resolution images were obtained with smaller cell aggregates. In these myceloids we observed irregular peptidoglycan synthesis, with most staining associated with adjacent poles of contiguous non-separated cells, the sites of joined cells being evident as cell envelope constrictions in the corresponding differential interference contrast images (as indicated by black arrows, S5A Fig). In many long cells (of average 1.1 μm length, examples indicated by white arrows in the corresponding overlay image), there was no observable nascent peptidoglycan in the midcell region. A similar picture emerged when salt-induced myceloids of the wild-type were examined. Cultures were grown to early log phase (20 h) in LB amended to a final concentration of 0.57 M NaCl. Fluo-vancomycin staining revealed irregular patterns of nascent peptidoglycan, with a reduced frequency of midcell peptidoglycan synthesis evident in longer cells (indicated by white arrows in the overlay image, S5B Fig). The myceloids of the strain expressing TreC_Ec_ (strain Ar0012) also showed evidence of reduced synthesis of peptidoglycan at the midcell with evidence of longer cells (indicated by white arrows in S5C Fig).

In addition, to examine how salt stress affected the transition from bacillary to myceloid growth of the wild-type in a time-course, cultures were grown to early log-phase in LB and subsequently in LB amended to a final concentration of 0.57 M NaCl, sampled at successive time-points and stained with fluo-vancomycin. In non-amended medium, after 3h, the proportion of cells scored with midcell peptidoglycan synthesis was 62% (n = 480), whereas with salt-stress, the percentage was reduced to 49% (n = 280). In addition, in cells from non-amended medium, we also observed one or two foci of peptidoglycan synthesis in the lateral walls in 39.4% of cells (n = 513); these foci are likely sites for growth of branches, consistent with the tendency for the wild-type to form occasional emerging branches as observed in SEM images (Fig 1C). After 3 h salt-stress, there was an increase to 66.5% (n = 524) of the proportion of cells with foci of nascent peptidoglycan in the lateral walls.

### OtsA can self-assemble into networks and assembly is promoted by trehalose-6-phosphate

Using dynamic light scattering (DLS), we examined the native state of *Arthrobacter* OtsA expressed and purified from *E*. *coli*. This revealed two populations of the protein: smaller assemblies with an average diameter of 68.86 nm and much larger assemblies with an average diameter of 1738.42 nm (Fig 4A). The protein was reanalyzed after denaturing the multimeric forms in 4 M urea followed by dialysis. If all urea was removed prior to DLS, during the dialysis process (16 h) all the protein self-assembled into two populations of multimeric forms with similar average diameters to those of assemblies prior to denaturation (S6 Fig). After denaturing in 4M urea and subsequent dilution to a final concentration of 1M urea, a much slower self-assembly process could be monitored by DLS in real-time (Fig 4B). We used these conditions to then ask if T6P can promote OtsA self-assembly. We observed that increasing concentrations of this metabolite had a dramatic effect on promoting the rate of OtsA polymerization (Fig 4B). Prior to addition of T6P, the average diameter of OtsA was 64.86 nm. After polymerization of OtsA promoted by 500 μM T6P (a physiologically relevant concentration–see above), there was only one population of the protein detected consisting of large assemblies with an average diameter of 2745.58 nm. The implication is that T6P can promote the assembly of OtsA into large protein networks.

As 1mM MgCl_2_ was used in these assays and magnesium ions are required for OtsA enzyme activity [28], we then examined whether magnesium ions have a role in the self-assembly of OtsA. Purified OtsA was denatured as described above and then dialysed against a phosphate buffer containing 1M urea and no magnesium ions. The protein was then incubated with between 0 and 1mM MgCl_2_ and assembly monitored in real time using DLS. Little or no assembly was observed in the absence of the ion, whereas addition of 1mM or greater MgCl_2_ promoted assembly (Fig 4C), although the maximum assembly was much less than that observed in the presence of T6P (Fig 4B).

We also used DLS to analyse the native state of OtsA_R36A_, revealing protein structures of 26.5 nm average diameter (S7A Fig). Moreover, addition of T6P failed to promote self-assembly of OtsA_R36A_ (Fig 4B). Consequently, we inferred that the lack of any morphogenetic activity of OtsA_R36A_ is not simply due to its loss of enzyme activity but presumably because the amino substitution also affects the protein’s tertiary structure and its ability to both interact with T6P and form large assemblies. DLS analysis of OtsA_*Ec*_ indicated protein structures with a range of sizes, and a modal diameter of approximately 7 nm (S7A Fig). No increase in diameter was observed after addition of T6P (Fig 4B).

A feature of the *Arthrobacter* OtsA is its enzymatic activity at low temperatures due to a very flexible ‘N-loop’ containing the active site [25], a characteristic that distinguishes the protein from its *E*. *coli* counterpart. To test if this flexible N-loop affects assembly formation, we purified and tested the assembly of two more OtsA proteins, OtsA_A3mu_ and OtsA_Ecmu_, which have, respectively, the *E*. *coli* N-loop replacing that of the *Arthrobacter* protein and *vice versa*. Whereas OtsA_A3mu_ retained the ability to polymerize, albeit less efficiently, OtsA_Ecmu_ behaved like OtsA_*Ec*_ with no evidence for self-assembly (S8 Fig). Consequently, we inferred that the N-loop alone is insufficient to promote self-assembly.

We used transmission electron microscopy (TEM) to examine different states of assembly of OtsA. As urea will interfere with negative staining, we chose to dialyse purified OtsA in phosphate buffer lacking magnesium ions. This resulted in depolymerization as evident in the sizes of imaged protein structures which had an average diameter of approximately 60 nm (Fig 4D), consistent with the dimensions of OtsA depolymerized after urea treatment as determined by DLS. Negative-stained OtsA_*Ec*_, purified the same way, was most abundant as structures of approximately 10 nm diameter (S7B Fig), again consistent with the size of protein structures determined by DLS analysis (see above). TEM of OtsA_R36A_ revealed structures of approximately 25 nm diameter (S7B Fig), consistent with the DLS data for this protein.

**Fig 5:**
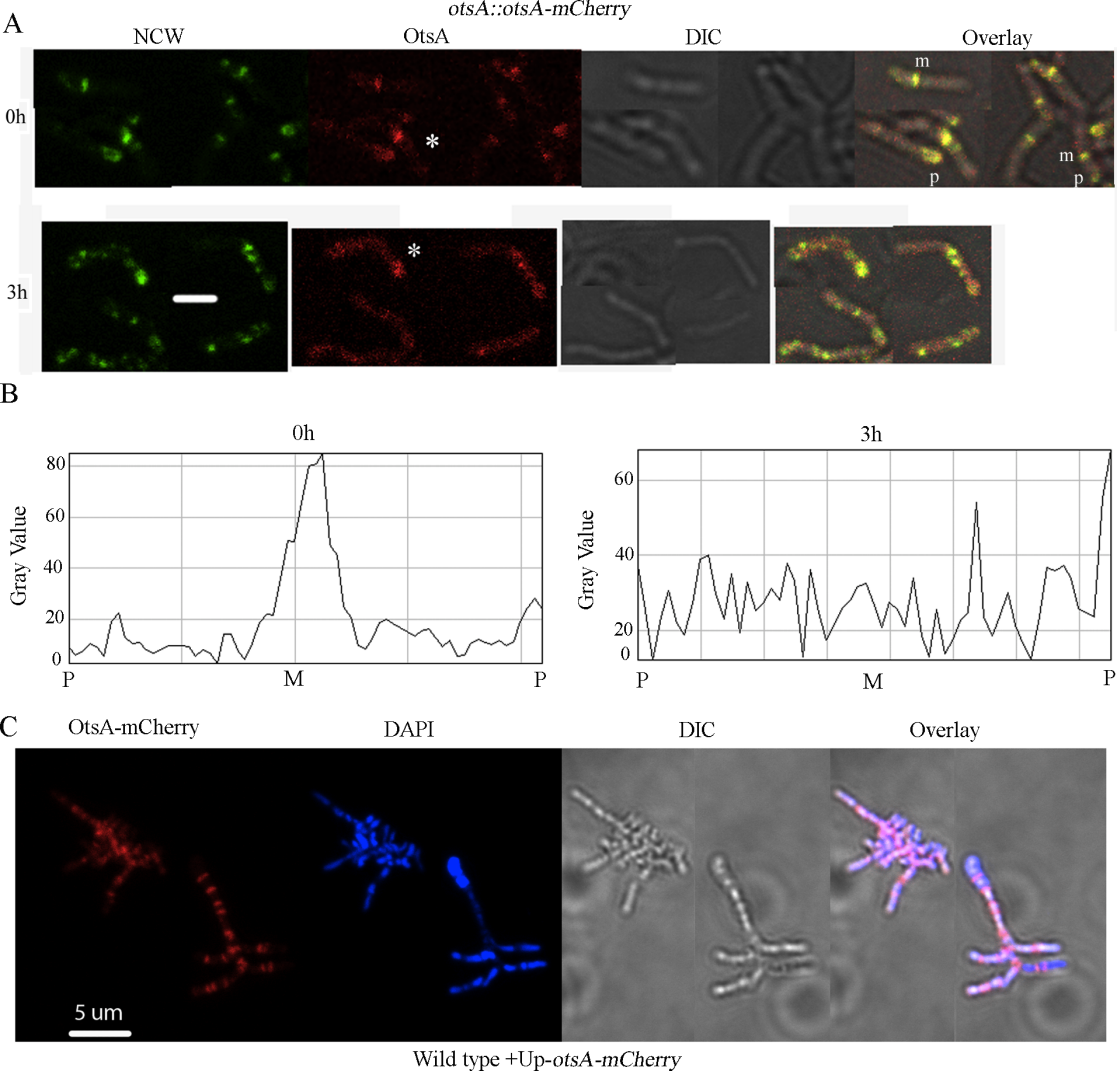
Localisation of OtsA at the midcell region is perturbed by osmotic stress. (A) Fluo-vancomycin staining of nascent cell walls (NCW, green) and OtsA::mCherry (red) were visualised by fluorescence microscopy. The strain Ar0007 (*otsA*::*otsA*-mCherry) was grown in LB to early log-phase (0 h) and subsequently in medium amended to a final concentration of 0.57 M NaCl (3 h). ‘m’ and ‘p’ denote midcell and pole, respectively, in the overlay images. Asterisks in the images visualizing OtsA-mCherry denote cells in which fluorescence distribution was quantified at 0 and 3 h. (B) Fluorescence intensities in these cells was plotted graphically: the x-axis corresponds to the distance from one pole (P), across the midcell region (M), to the opposite pole. (C) Representative fluorescence microscopy images of fluo-vancomycin staining of nascent cell walls (green), DAPI staining of nucleoids (blue), the DIC image and the corresponding merged overlay images are shown for strain Ar0006 (overexpressed *otsA*-mCherry). Scale bars in microscopy images are equivalent to 2 um.

We then imaged OtsA after addition of magnesium ions alone or combined with T6P. After incubation for 30 min with 0.1mM MgCl_2_ we observed the appearance of branched protein filaments of varying lengths, up to approximately 200 nm in length (Fig 4D). After 30 min incubation with 1 mM MgCl_2_ and 500 μM T6P, very large assemblies could be observed but only with low resolution using negative staining. Consequently, we used positive staining to obtain images of better resolution, as exemplified in Fig 5D, indicating assembly of the protein into large networks of greater than 2000 nm diameter.

### OtsA localizes to the midcell and poles in non-stressed cells

To examine localization of OtsA *in vivo*, a C-terminal translational fusion with mCherry was expressed in *Arthrobacter* A3 using the native *otsA* promoter sequence and a single-copy gene fusion integrated at the chromosomal *otsA* locus (strain Ar0007). Cells expressing the fusion protein grew normally. In addition, morphogenetic functionality was indicated both by the ability of the fusion protein expressed under control of the native promoter to restore normal snap-division growth to the Δ*otsA* mutant (strain Ar0116; S3 Fig) and by the promotion of the characteristic multiple-septation phenotype in long, enlarged cells when the fusion protein was overexpressed (strain Ar0006, Fig 5B). In Ar0007 cells grown in LB, the majority of the protein assembled at the midcell region (indicated as ‘m’ in the overlay image, Fig 5A) and some at the cell poles (indicated as ‘p’ in the overlay image, Fig 5A), co-localizing at sites of peptidoglycan synthesis as revealed by fluo-vancomycin staining of the same cells (Fig 5A). To examine if osmotic stress affects protein localisation, strain Ar0007 expressing OtsA::mCherry was grown to early log phase in LB, which was then amended to a final concentration of 0.57 M NaCl. After 3 h salt stress, we observed a diffuse distribution of OtsA throughout the cells (Fig 5A), some colocalising with sites of peptidoglycan synthesis at the cell poles, but no longer localized at the midcell region. When the fusion protein was overexpressed it clearly localized to the sites of multiple septum formation, again indicative of a morphogenetic function (Fig 5B).

## Discussion

Coordinating trehalose concentration and morphology requires that pleomorphic *Arthrobacter* cells can detect osmotic stress and communicate this information to the cell division apparatus. Here we describe an unexpected role for the trehalose synthase protein OtsA, which doubles as a morphogenetic protein, acting as a direct link between trehalose synthesis and cell morphology, and effecting the transition from bacillary growth to the development of myceloids. A product of the OtsA enzyme, T6P, can function as a signaling proxy for osmotic stress but is insufficient itself to direct changes in cell morphology as evidenced from the lack of any morphogenetic function of the otherwise enzymatically active OtsA_Ec_.

*In vitro*, OtsA can self-assemble to form elaborate protein networks, this assembly being promoted by T6P. *In vivo*, when cells are growing in low medium osmolarity, we hypothesise that these protein networks have a morphogenetic cytoskeletal function in promoting normal cytokinesis leading to a bacillary growth-style. Indeed, in non-stressed cells the protein assembles at the midcell and poles, consistent with this hypothesis. We have analyzed the morphological outcomes of various permutations of the genetic background of *Arthrobacter* that affect either or both intracellular T6P and OtsA concentrations (Table 1). Overexpression of OtsA, resulting in increased T6P, leads to increased formation of septa and loss of coordination of cytokinesis. Moreover, the overexpressed protein localizes at the multiple sites of septum formation in filamentous cells. But an increase in intracellular T6P, due to overexpression of OtsA_Ec,_ is insufficient itself to cause aberrant cytokinesis. Salt stress leads to a depletion of T6P and this likely affects the dynamics of OtsA self-assembly *in vivo*, resulting in the observed diffuse cytoplasmic distribution of the protein in salt-stressed cells and the reduction of peptidoglycan assembly at the midcell, but increasing the likelihood of the emergence of branches from lateral cell walls. These effects, coupled with a reduction in snap-division frequency, presumably lead to the growth of myceloids. Additional evidence that T6P can act as an intracellular proxy for salt stress comes from experimentally depleting this metabolite by expression of *E*. *coli* T6P hydrolase. A consequence of this depletion is that the strain can only grow with a myceloid morphology, despite expressing normal levels of OtsA.

**Table 1:** Morphological outcomes of manipulating OtsA and T6P levels.

Medium osmolarity	Genetic background	T6P concentration	Predicted OtsA assembly *in vivo*	Outcome
Low	Wild-type	Normal	Normal	Bacillary growth
High	Wild-type	Reduced	Reduced	Myceloid growth
Low	Δ*otsA*	Very reduced	Absent	Myceloid growth
Low	Δ*otsA* + *otsA*_*Ec*_	Normal	Absent	Myceloid growth
Low	Δ*otsA* + *otsA*_*R36A*_	Reduced	Absent	Myceloid growth
Low	Wild-type + *treC*_*Ec*_	Very reduced	Reduced	Myceloid growth
Low	Wild-type + up-*otsA*	Increased	Increased	Irregular cytokinesis
Low	Wild-type + up-*otsA*_*Ec*_	Increased	Normal	Bacillary growth
Low	Wild-type + up-*otsA*_*R36A*_	Normal	Normal	Bacillary growth

The psychrotrophic nature *Arthrobacter* A3 is to an extent due to its ability to accumulate trehalose as a cryoprotectant, which in turn is due to the enzymatic activity of OtsA at low temperatures [25]. This activity is due to a very flexible ‘N-loop’ containing the active site [25], a key feature that distinguishes the protein from its *E*. *coli* counterpart. However, exchanging the N-loops of the *Arthrobacter* and *E*. *coli* proteins indicated that this sequence alone is not responsible for the self-assembly characteristic of the former, although the *Arthrobacter* protein with an *E*. *coli* N-loop is less proficient at self-assembly compared to its wild-type counterpart. In the genetic backgrounds in which either OtsA_R36A_ or OtsA_Ec_ are overexpressed and T6P levels are normal or increased, these proteins have no effect on cytokinesis. The lack of *in vivo* morphogenetic and *in vitro* self-assembly activities of either OtsA_R36A_ or OtsA_Ec_ can be rationalized in part by the active site mutation of the former and reduced N-loop flexibility of the latter, both of which likely affect T6P binding. We are currently comparing the structural properties of these variant proteins to identify other features that contribute to the ability of *Arthrobacter* OtsA to self-assemble.

Our analysis of published *Arthrobacter* genome sequences indicates that these bacteria lack typical actin-like or intermediate filament cytoskeletal proteins found in other bacteria, including some other actinobacteria. The evolutionary recruitment of the principle biosynthetic enzyme involved in synthesis of the osmoprotectant trehalose to a function that regulates morphology in response to osmotic stress is an intriguing adaptation to coping with extreme environments. This adds to a few other known examples of biosynthetic enzymes co-opted for morphogenetic roles in bacteria. The primary enzyme involved in CTP synthesis, CtpS, from *Caulobacter*, *E*. *coli* and several eukaryotic species can self-assemble into linear filaments [32–34] and in *Caulobacter* this protein has a role in determining cell shape [32]. In the same bacterium, a NAD(H)-binding oxidoreductase, KidO, can inhibit Z-ring formation [35], linking cell division with metabolic status. In *Bacillus subtilis* the membrane-associated glucosyltransferase UgtP, involved in glycolipid biosynthesis, acts as a metabolic sensor governing cell size. During growth in rich media, when *ugtP* expression is upregulated, the enzyme localises to the midcell division site and inhibits Z-ring formation at the midcell [36]. In *E*. *coli*, a non-homologous glucosyltransferase OpgH, an integral inner membrane protein that is functionally analogous to UgtP of *B*. *subtilis* in linking cell size with central metabolism, is believed to inhibit Z-ring formation by a different mechanism involving sequestering FtsZ [37]. In these latter two examples, the bacteria utilise UDP-glucose as an intracellular signal and proxy for nutrient availability. In contrast to these examples, in *Arthrobacter* the OtsA glucosyltransferase doubles as a morphogenetic protein and determine cell morphology in response to an environmental signal.

Although several other bacterial species are known to exhibit morphological plasticity as a stress survival strategy, switching from a rod-shaped to a filamentous morphology [38], the mechanism for this transition, when known, is quite different. In *E*. *coli*, the product of an SOS-induced gene, SulA, binds to FtsZ monomers, inhibiting polymerization [39]. In older *Caulobacter* cells, depletion of FtsZ leads to filamentation [40]. It will be of interest to determine if OtsA in *Arthrobacter* interacts with FtsZ and to investigate the properties of OtsA from other actinobacteria, including *M*. *tuberculosis* which undergoes filamentation in macrophages [41].

## Materials and methods

### Bacterial strains, plasmids, and cell growth

The growth conditions for *Arthrobacter* strains and *E*. *coli* cultures are given in detail in the Supplemental protocols. The list of all relevant strains and plasmids is provided in S1 Materials and Methods. Details of how each plasmid and strain were constructed, and the primers used for amplification and cloning of DNA sequences are also provided in the Supplemental protocols.

### Fluorescence and immunofluorescence microscopy

All strains were analyzed in exponential growth phase unless otherwise stated. Nascent cell walls were stained using fluo-vancomycin as described previously [42]. Bacterial cells were stained by DAPI (0.1ug/ml, PBS), vancomycin (1ug/ml, PBS) and fluo-vancomycin (1ug/ml, PBS) for 20min. Cultures were then washed in PBS, and suspended in 1.6% formaldehyde (in PBS) and left on ice for 1 hr. Treated cells were distributed on microscope slides that had been treated with 0.1% (wt/vol) poly-L-lysine (Sigma). Images were acquired on a confocal laser scanning microscope (Olympus FV1000).

### Purification of proteins, immunoblot analysis, and coimmunoprecipitation analysis

The purification of proteins, immunoblotting and coimmunoprecipitation analysis were carried out as described previously [24, 25].

### OtsA assembly assays

The assembly of OtsA was monitored in real-time with a dynamic light scattering assay using a Brookhaven Instruments BI-200SM system (USA). The wavelength of the stable argon ion laser was 532 nm. The assay was performed at 20°C. OtsA was denaturated in 4 M urea for 5 min and then diluted in polymerization buffer O (20mM Tris HCl, pH 7.5, 1 mM MgCl_2_), with different concentrations of T6P, to 10 uM final concentration of OtsA and 1M of urea. To measure the effect of magnesium ions, MgCl_2_ was excluded from buffer O. The intensity of scattered light was measured at an angle of 90°.

### Electron microscopy

Negative staining electron microscopy was used to visualize OtsA. Carbon-coated copper grids (400 mesh, Electron Microscopy Sciences) were glow discharged for 5 s before use. Before applying OtsA, a drop of 0.2 mg/ml cytochrome *c* was pipetted onto the carbon, incubated for 30 s, and then blotted with filter paper. A drop of OtsA solution was then applied to the carbon and incubated for 10 s before the excess was blotted. The grid was immediately rinsed with 3–4 drops 2% uranyl acetate, blotted, and air-dried. For positive staining, 5 ul of protein (10 μM) were placed on carbon coated grids, incubated for 2 min, washed in buffer A (20 mM sodium phosphate, pH 7.5 and 20 mM NaCl), and stained with 2% uranylacetate for 30 s. Protein were visualized and photographed using a Tecnai-G2-F30 electron microscope. To image cells, exponentially growing *Arthrobacter* strains were fixed in 4% paraformaldehyde, 2.5% glutaraldehyde (PBS, PH 7.4), at 25°C for 2 h. SEM of whole cells were carried out as described previously [43].

### Trehalose-6-phosphate and trehalose assays

Trehalose-6-phophate phosphatase was obtained from *E*. *coli* as described [44]. To measure trehalose-6-phosphate, cells were broken using ultrasonication at 4 ^0^C and extracts prepared and assayed as described [45]. For each strain, four replicate assays were conducted. Trehalose was assayed as previously described [24].

## Supporting information

S1 Fig Determination of intracellular trehalose concentrations. Intracellular concentrations were obtained from cells grown to mid-log phase and standardized with respect to wet weights. (A) Intracellular trehalose concentration of Ar0001 (wt), Ar0002 (ΔotsA), Ar0008 (wt + treFEc) and Ar0112 (ΔotsA + otsAEc) grown in LB medium; (B) Intracellular trehalose concentrations for Ar0001 (wt) grown in LB and LB supplemented with 0.4 M NaCl. (C) Intracellular trehalose concentrations for Ar0003 (wt + empty vector) and Ar0010 (wt + up-otsAEc) grown in LB. (D) Intracellular trehalose concentrations for Ar0001 (wt), Ar0002 (ΔotsA) grown in LB and Ar0002 (ΔotsA) grown in LB with 4 mM trehalose. The values plotted represent averages from 4 independent experiments.
(DOCX)

S2 Fig The percentage of cell aggregates with circumference >4 um in early log-phase cultures of wild-type (Ar0001), ΔotsA (Ar0002), wild-type + treFEc (Ar0008) grown in LB medium and wild-type (Ar0001) grown in LB medium amended to a final concentration of 0.57 M NaCl. (DOCX)

S3 Fig Growth curves of strains Ar0111 (Δ*otsA* + *P_otsA_-otsA*), Ar0112 (Δ*otsA* + *P_otsA_-otsA_Ec_*), Ar0113 (ΔotsA + *P_otsA_-otsB*), Ar0114 (Δ*otsA* + *P_otsA_-dsbA*), Ar0115 (ΔotsA + *P_otsA_-otsA*_R36A_), Ar0116 (ΔotsA + *P_otsA_-otsA*::mCherry) grown in LB. (DOCX)

S4 Fig Strain Ar0003 (Wild type strain, 1); Ar0111 (Δ*otsA* + *P*_*otsA*_-*otsA*, 2); Ar0002 (Δ*otsA*, 3); Ar0004 (wild-type + up-*otsA*, 4) Ar0010 (wild-type + up-*otsA*_*EC*_, 5); Ar0011 (wild-type + up-*otsA*_*R36A*_, 6) were grown on minimal medium (A), minimal medium+ 4 mM trehalose (B), minimal medium + 0.57 M NaCl (C), minimal medium + 4mM trehalose + 0.57 M NaCl (D).
(DOCX)

S5 Fig Reduced septum formation associated with growth of myceloids. NCW, DAPI, DIC and merged images of representative early log-phase myceloids of (A) strain Ar0002 (ΔotsA) grown in LB; (B) strain Ar0003 (wild-type with empty vector) grown in LB amended to a final concentration of 0.57 M NaCl; (C) strain Ar0012 (wild-type with treCEc) grown in LB. Black arrows in the DIC image (A) indicate the joints of non-separated cells and white arrows in the merged images indicate representative long cells. Scale-bars represent 2 μm.
(DOCX)

S6 Fig Sizes of OtsA protein multimers previously denatured in 4M urea and then extensively dialysed to remove all urea, measured by dynamic light scattering. (DOCX)

S7 Fig Analysis of protein multimers of OtsAEc and OtsAR36A. (A) Sizes and abundance of multimeric structures as determined by dynamic light scattering. (B) Negative-stained TEM images of the respective protein structures.
(DOCX)

S8 Fig Polymerization of variant OtsA proteins. (DOCX)

S1 Materials and Methods (DOCX)
