# Supplementary material for: A trehalose biosynthetic enzyme doubles as an osmotic stress sensor to regulate bacterial morphogenesis
Source: PLoS Genet. 2017 Oct 30;13(10):e1007062. doi: 10.1371/journal.pgen.1007062 (PMC5685639; doi:10.1371/journal.pgen.1007062)
Supplement: S1 Fig — Intracellular concentrations were obtained from cells grown to mid-log phase and standardized with respect to wet weights. (A) Intracellular trehalose concentration of Ar0001 (wt), Ar0002 (ΔotsA), Ar0008 (wt + treFEc) and Ar0112 (ΔotsA + otsAEc) grown in LB medium; (B) Intracellular trehalose concentrations for Ar0001 (wt) grown in LB and LB supplemented with 0.4 M NaCl. (C) Intracellular trehalose concentrations for Ar0003 (wt + empty vector) and Ar0010 (wt + up-otsAEc) grown in LB. (D) Intracellular trehalose concentrations for Ar0001 (wt), Ar0002 (ΔotsA) grown in LB and Ar0002 (ΔotsA) grown in LB with 4 mM trehalose. The values plotted represent averages from 4 independent experiments. (DOCX) [file pgen.1007062.s001.docx]

Supplemental Fig S1





**Fig S1: Determination of intracellular trehalose concentrations. Intracellular concentrations were obtained from cells grown to mid-log phase and standardized with respect to wet weights. (A) Intracellular trehalose concentration of Ar0001 (wt), Ar0002 (ΔotsA), Ar0008 (wt + treFEc) and Ar0112 (ΔotsA + otsAEc) grown in LB medium; (B) Intracellular trehalose concentrations for Ar0001 (wt) grown in LB and LB supplemented with 0.4 M NaCl. (C) Intracellular trehalose concentrations for Ar0003 (wt + empty vector) and Ar0010 (wt + up-otsAEc) grown in LB. (D) Intracellular trehalose concentrations for Ar0001 (wt), Ar0002 (ΔotsA) grown in LB and Ar0002 (ΔotsA) grown in LB with 4 mM trehalose. The values plotted represent averages from 4 independent experiments.**
