# Supplementary material for: A trehalose biosynthetic enzyme doubles as an osmotic stress sensor to regulate bacterial morphogenesis
Source: PLoS Genet. 2017 Oct 30;13(10):e1007062. doi: 10.1371/journal.pgen.1007062 (PMC5685639; doi:10.1371/journal.pgen.1007062)
Supplement: S2 Fig — (DOCX) [file pgen.1007062.s002.docx]

Fig S2: The percentage of cell aggregates with circumference >4 um in early log-phase cultures of wild-type (Ar0001), ΔotsA (Ar0002), wild-type + treFEc (Ar0008) grown in LB medium and wild-type (Ar0001) grown in LB medium amended to a final concentration of 0.57 M NaCl.
