# Supplementary material for: A trehalose biosynthetic enzyme doubles as an osmotic stress sensor to regulate bacterial morphogenesis
Source: PLoS Genet. 2017 Oct 30;13(10):e1007062. doi: 10.1371/journal.pgen.1007062 (PMC5685639; doi:10.1371/journal.pgen.1007062)
Supplement: S3 Fig — (DOCX) [file pgen.1007062.s003.docx]

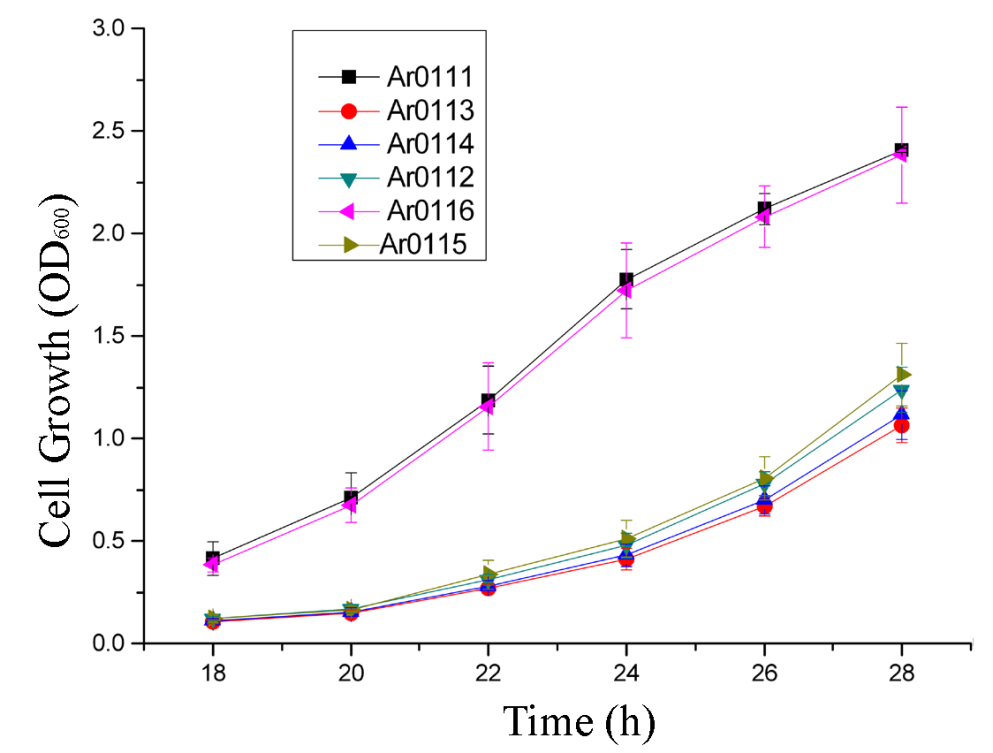


**Fig S3: Growth curves of strains Ar0111 (Δ*otsA* + *P_otsA_*-*otsA*), Ar0112 (Δ*otsA* + *P_otsA_*-*otsA_Ec_*), Ar0113 (ΔotsA + *P_otsA_*-*otsB*), Ar0114 (Δ*otsA* + *P_otsA_*-*dsbA*), Ar0115 (ΔotsA + *P_otsA_*-*otsA*_R36A_), Ar0116 (ΔotsA + *P_otsA_*-*otsA*::mCherry) grown in LB.**
