# Supplementary material for: A trehalose biosynthetic enzyme doubles as an osmotic stress sensor to regulate bacterial morphogenesis
Source: PLoS Genet. 2017 Oct 30;13(10):e1007062. doi: 10.1371/journal.pgen.1007062 (PMC5685639; doi:10.1371/journal.pgen.1007062)
Supplement: S4 Fig — Strain Ar0003 (Wild type strain, 1); Ar0111 (ΔotsA + PotsA-otsA, 2); Ar0002 (ΔotsA, 3); Ar0004 (wild-type + up-otsA, 4) Ar0010 (wild-type + up-otsAEC, 5); Ar0011 (wild-type + up-otsAR36A, 6) were grown on minimal medium (A), minimal medium+ 4 mM trehalose (B), minimal medium + 0.57 M NaCl (C), minimal medium + 4mM trehalose + 0.57 M NaCl (D). (DOCX) [file pgen.1007062.s004.docx]

Supplemental Figure 4





**Fig S4: Strain Ar0003 (Wild type strain, 1); Ar0111 (Δ*otsA* + *P_otsA_*-*otsA*, 2); Ar0002 (Δ*otsA*, 3); Ar0004 (wild-type + up-*otsA*, 4) Ar0010 (wild-type + up-*otsA_EC_*, 5); Ar0011 (wild-type + up-*otsA_R36A_*, 6) were grown on minimal medium (A), minimal medium+ 4 mM trehalose (B), minimal medium + 0.57 M NaCl (C), minimal medium + 4mM trehalose + 0.57 M NaCl (D).**
