# Supplementary material for: A trehalose biosynthetic enzyme doubles as an osmotic stress sensor to regulate bacterial morphogenesis
Source: PLoS Genet. 2017 Oct 30;13(10):e1007062. doi: 10.1371/journal.pgen.1007062 (PMC5685639; doi:10.1371/journal.pgen.1007062)
Supplement: S5 Fig — NCW, DAPI, DIC and merged images of representative early log-phase myceloids of (A) strain Ar0002 (ΔotsA) grown in LB; (B) strain Ar0003 (wild-type with empty vector) grown in LB amended to a final concentration of 0.57 M NaCl; (C) strain Ar0012 (wild-type with treCEc) grown in LB. Black arrows in the DIC image (A) indicate the joints of non-separated cells and white arrows in the merged images indicate representative long cells. Scale-bars represent 2 μm. (DOCX) [file pgen.1007062.s005.docx]

Supplemental Fig S5


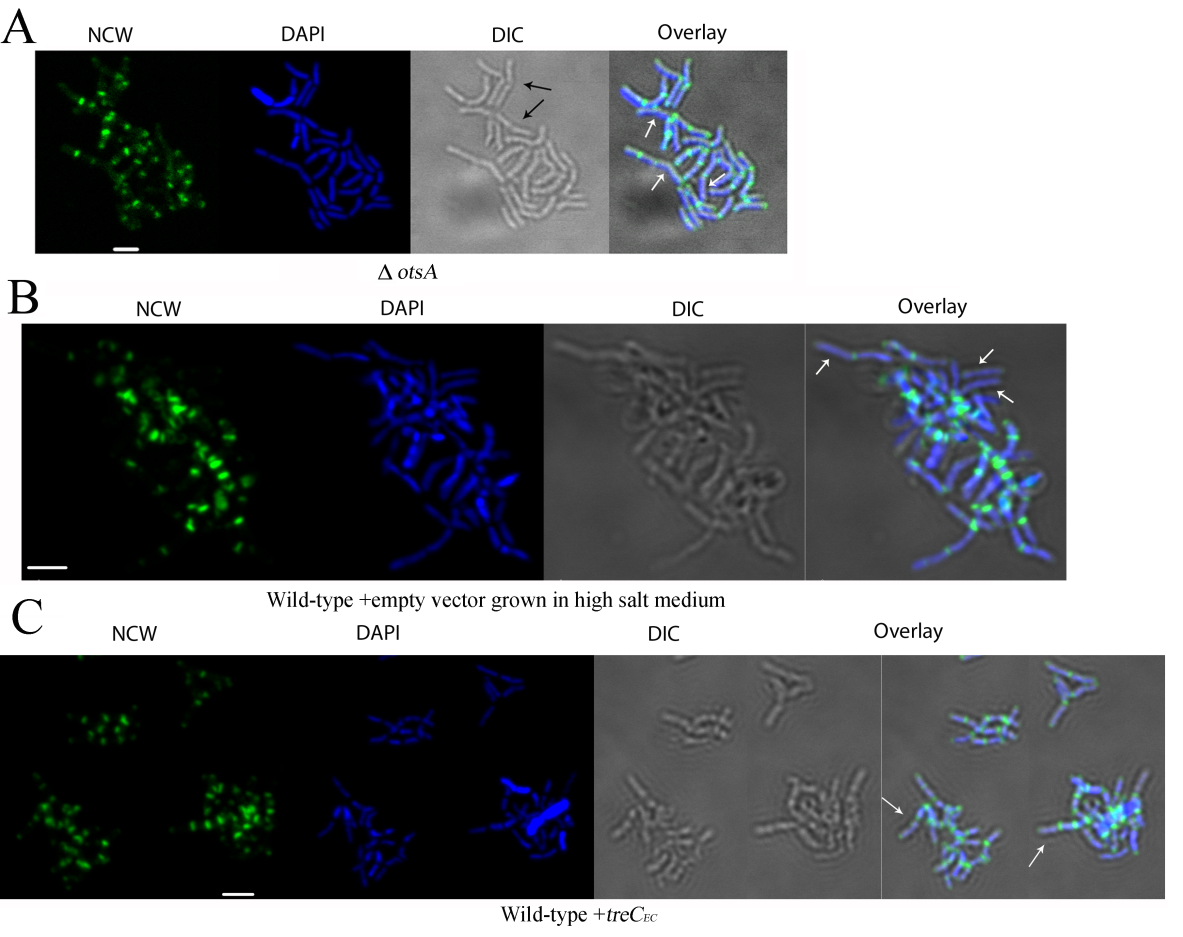


Fig S5: **Reduced septum formation associated with growth of myceloids**

NCW, DAPI, DIC and merged images of representative early log-phase myceloids of (A) strain Ar0002 (Δ*otsA*) grown in LB; (B) strain Ar0003 (wild-type with empty vector) grown in LB amended to a final concentration of 0.57 M NaCl; (C) strain Ar0012 (wild-type with *treC_Ec_*) grown in LB. Black arrows in the DIC image (A) indicate the joints of non-separated cells and white arrows in the merged images indicate representative long cells. Scale-bars represent 2 μm.
