# Supplementary material for: A trehalose biosynthetic enzyme doubles as an osmotic stress sensor to regulate bacterial morphogenesis
Source: PLoS Genet. 2017 Oct 30;13(10):e1007062. doi: 10.1371/journal.pgen.1007062 (PMC5685639; doi:10.1371/journal.pgen.1007062)
Supplement: S6 Fig — (DOCX) [file pgen.1007062.s006.docx]

**
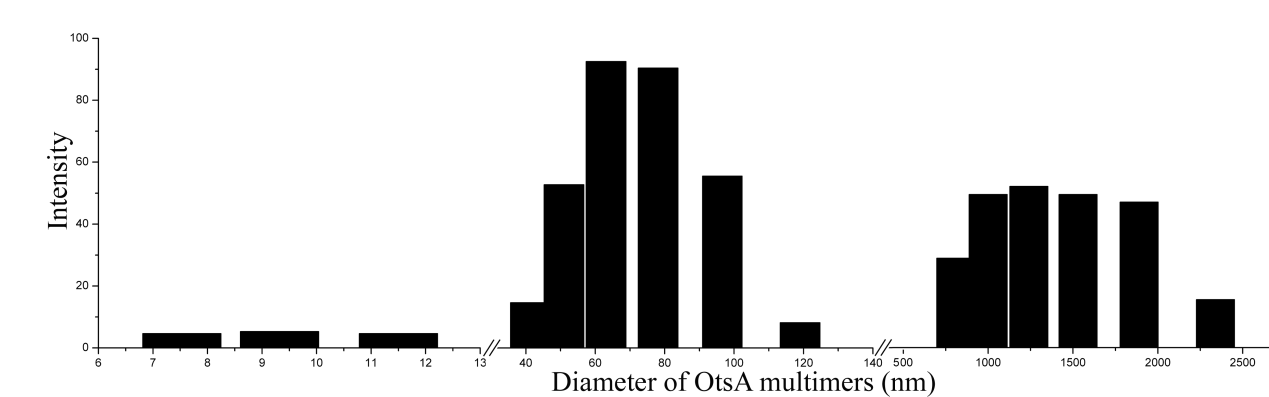
**

**Fig S6:** **Sizes of OtsA protein multimers previously denatured in 4M urea and then extensively dialysed to remove all urea, measured by dynamic light scattering.**
