# Supplementary material for: A trehalose biosynthetic enzyme doubles as an osmotic stress sensor to regulate bacterial morphogenesis
Source: PLoS Genet. 2017 Oct 30;13(10):e1007062. doi: 10.1371/journal.pgen.1007062 (PMC5685639; doi:10.1371/journal.pgen.1007062)
Supplement: S7 Fig — (A) Sizes and abundance of multimeric structures as determined by dynamic light scattering. (B) Negative-stained TEM images of the respective protein structures. (DOCX) [file pgen.1007062.s007.docx]

**Supplemental Figure 7**

**
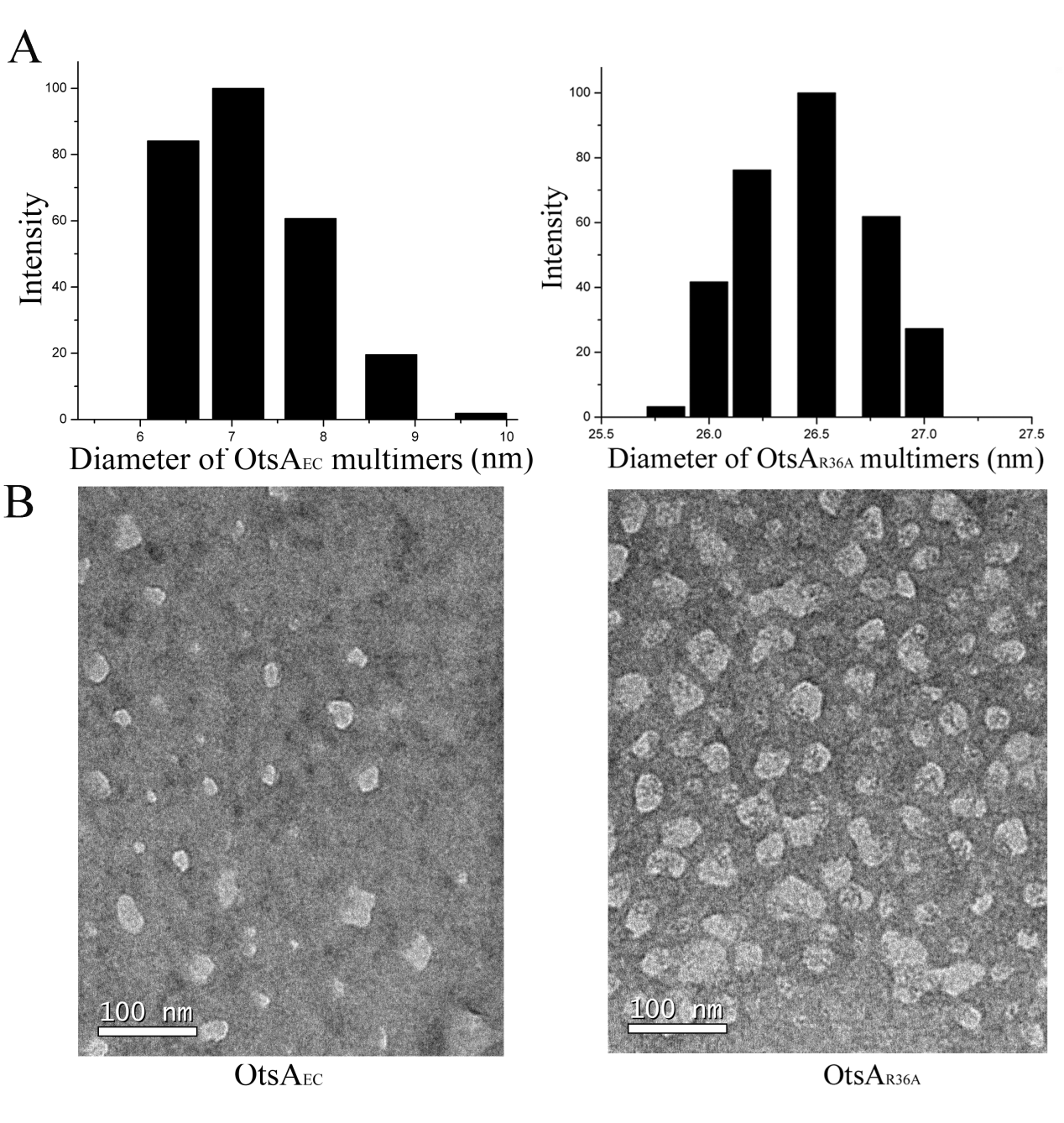
**

**Fig S7: Analysis of protein multimers of OtsA_Ec_ and OtsA_R36A_. (A) Sizes and abundance of multimeric structures as determined by dynamic light scattering. (B) Negative-stained TEM images of the respective protein structures.**
