# Supplementary material for: A trehalose biosynthetic enzyme doubles as an osmotic stress sensor to regulate bacterial morphogenesis
Source: PLoS Genet. 2017 Oct 30;13(10):e1007062. doi: 10.1371/journal.pgen.1007062 (PMC5685639; doi:10.1371/journal.pgen.1007062)
Supplement: S8 Fig — (DOCX) [file pgen.1007062.s008.docx]

Supplemental Fig 8


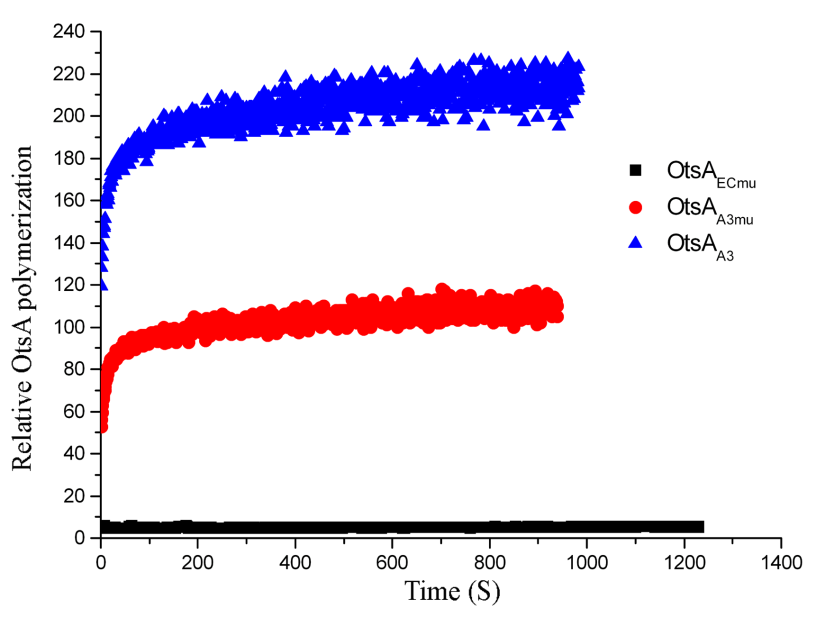


Fig S8. Polymerization of variant OtsA proteins. Self-assembly of OtsA (= OtsA_A3_), OtsA_A3mu_ and OtsA_Ec_mu in 1M urea and 1mM MgCl2 with 500 μM T6P was measured using dynamic light scattering.
