# Supplementary material for: A trehalose biosynthetic enzyme doubles as an osmotic stress sensor to regulate bacterial morphogenesis
Source: PLoS Genet. 2017 Oct 30;13(10):e1007062. doi: 10.1371/journal.pgen.1007062 (PMC5685639; doi:10.1371/journal.pgen.1007062)
Supplement: S1 Materials and Methods — (DOCX) [file pgen.1007062.s009.docx]

**Supplemental Materials and Methods**

**Table A: Plasmids**

| **Name** | **Relevant description** | **Reference** |
| --- | --- | --- |
| **pAIP1** | Integrative suicide vector based on pART2 carrying *otsA*-*mCherry* under control of *P_otsA_* | This work |
| **pART2** | Medium copy number plasmid carrying the strong promoter of *hdnO*, Kan^r^ | ([Sandu et al., 2005](#_ENREF_5)) |
| **pART2-flag-*otsA*** | pART2 carrying Flag-*otsA* under control of *P_hdnO_* | (Chen et al., 2011) |
| **pART2-flag-*otsA*_R36A_** | pART2 carrying Flag-*otsA*_R36A_ under control of *P_hdnO_* | This work |
| **pART2-*otsA_Ec_*** | pART2 carrying *E.coli* *otsA* under control of *P_hdnO_* | This work |
| **pART2-*treC_Ec_*** | pART2 carrying *treC_Ec_* under control of *P_hdnO_* | This work |
| **pART2-*treF_Ec_*** | pART2 carrying *E.coli* *treF* under control of *P_hdnO_* | This work |
| **pCO-*dsbA*** | Integrative suicide vector based on pSH152 carrying *dsbA* under control of native promoter | This work |
| **pCO-*otsA*** | Integrative suicide vector based on pSH152 carrying *otsA* under control of its native promoter | This work |
| **pART2-*otsA*-*mCherry*** | pART2 carrying *otsA*-*mCherry* under control of *P_hdnO_* | This work |
| **pCO-*otsA*_R36A_** | Integrative suicide vector based on pSH152 carrying *otsA*_R36A_ under control of *otsA* native promoter | This work |
| **pCO-*otsA_Ec_*** | Integrative suicide vector based on pSH152 carrying *otsA****_Ec_*** under control of *P_otsA_* | This work |
| **pCO-*otsB*** | Integrative suicide vector based on pSH152 carrying *otsB* under control of *P_otsA_* | This work |
| pCO-*otsA*-*mCherry* | Integrative suicide vector based on pSH152 carrying *otsA-mCherry* under control of *otsA* native promoter | This work |
| **pET28** | Expression vector used for C-term His-tag, Kan^r^ | Novagen |
| **pET28-*otsA*** | Plasmid expressing OtsA-His6 | This work |
| **pET28-*otsA_Ec_*** | Plasmid expressing *E.coli* OtsA-His6 | This work |
| **pET28-OtsA_ECmu_** | Plasmid expressing a fusion in which the N-loop sequence of OtsA replaced that of OtsA_Ec_ | This work |
| **pET42-OtsA_A3mu_** | Plasmid expressing a fusion in which the N-loop sequence of OtsA_Ec_ replaced that of OtsA | This work |
| **pSH152** | Integrative shuttle plasmid carrying hygromycin resistance gene, Hyg^r^ | (Mistry et al., 2008) |

**Table B: Strains**

| **Name** | **Relevant genotype** | **Reference** |
| --- | --- | --- |
| *Arthrobacter* |  |  |
| Ar0001 | Wild type strain, *Arthrobacter* strain A3 | (Chen et al., 2011) |
| Ar0002 | Ar0001 Δ*otsA* | (Chen et al., 2011) |
| Ar0003 | Ar0001/pART2 | (Chen et al., 2011) |
| Ar0004 | Ar0001/pART2-flag-*otsA* | (Chen et al., 2011) |
| Ar0006 | Ar0001/pART2-*otsA*-*mCherry* | This work |
| Ar0007 | Ar0001 *otsA*::*otsA*-*mCherry* | This work |
| Ar0008 | Ar0001/pART2-*treF_Ec_* | This work |
| Ar0010 | Ar0001/pART2-*otsA_Ec_* | This work |
| Ar0011 | Ar0001/pART2-flag-*otsA*_R36A_ | This work |
| Ar0012 | Ar0001/pART2-*treC_Ec_* | This work |
| Ar0111 | Ar0001 Δ*otsA* *P_otsA_*-*otsA* | This work |
| Ar0112 | Ar0001 Δ*otsA P_otsA_*-*otsA_EC_* | This work |
| Ar0113 | Ar0001 Δ*otsA* *P_otsA_*-*otsB* | This work |
| Ar0114 | Ar0001 Δ*otsA* *P_dsba_*-*dsbA* | This work |
| Ar0115 | Ar0001 Δ*otsA P_otsA_*-*otsA*_R36A_ | This work |
| Ar0116 | Ar0001 Δ*otsA P_otsA_*-*otsA-mCherry* | This work |
| *E. coli* strains |  |  |
| DH5α | Cloning strain | Invitrogen |
| BL21 | Protein expression strain | Promega |
| EC1001 | BL21/pET28-*otsA* | This work |
| EC1002 | BL21/pET28-*otsA_EC_* | This work |
| EC1003 | BL21/pET28-OtsAECmu | This work |
| EC1004 | BL21/pET42-OtsAA3mu | This work |
| EC2002 | DH5α/pART2-*otsA*-*mCherry* | This work |
| EC2003 | DH5α/pART2-*otsA_Ec_* | This work |
| EC2006 | DH5α/pART2-*treF_Ec_* | This work |
| EC2007 | DH5α/ pCO-*otsA* | This work |
| EC2008 | DH5α/ pCO-*otsA_Ec_* | This work |
| EC2009 | DH5α/ pCO-*otsB* | This work |
| EC2010 | DH5α/ pCO-*dsbA* | This work |
| EC2011 | DH5α/ pART2-flag-*otsA****_R36A_*** | This work |
| EC2012 | DH5α/ pART2-*treC_Ec_* | This work |

**Supplemental Protocols**

**Bacterial Strains and Growth Conditions**

*E. coli* DH5α was used for general cloning purposes and BL21 was used for protein expression. All *Arthrobacter* strains used in this study are derived from the wild-type strain A3, Ar0001 (Chen et al., 2011). Electro-competent cells of *Arthrobacter* were prepared as described (Zhang et al., 2011). *E. coli* strains were grown aerobically in Luria-Bertoni (LB) broth at 37 ^o^C. Arthrobacter strains were grown at 20 ^o^C in Luria-Bertoni broth or GAUZE’s Medium NO.1.

For *E. coli*, antibiotics were used at the following concentrations (μg/ml): ampicillin 100, kanamycin 50, hygromycin 100. For *Arthrobacter* kanamycin was used at 140 ug/ml and hygromycin 200 μg/ml.

**Construction of plasmids**

**pAIP1**: *P_otsA_-otsA* was PCR amplified from a genomic DNA preparation of *Arthrobacter* strain A3 using primers pAIPPotsA p1 and pAIPPotsA p2. mCherry was PCR amplified from a plasmid pNA585 (Ausmees et al., 2007) using primers pAIPPotsA p3 and pAIPPotsA p4. Fusion PCR was used to link *otsA* and *mCherry* together. The purified PCR product was digested with HindIII and BamHI and ligated into pART2 digested with the same enzymes, thus removing the origin of plasmid replication in *Arthrobacter*.

**pART2-flag-*otsA*_R36A_**: flag-*otsA*_R36A_ was PCR amplified from a plasmid DNA preparation of pART2-flag-*otsA* using primers Flag otsA p1, otsAR36A p2, otsAR36A p3 and Flag otsA p2. The purified PCR product was digested with BamHI and XbaI and ligated into pART2 digested with the same enzymes.

**pART2-*otsA_Ec_***: *otsA_Ec_* was PCR amplified from a genomic DNA preparation of DH5a using primers pART2otsAEC p1 and pART2 otsAEC p2. The purified PCR product was digested with BglII and XbaI and ligated into pART2 digested with the BamHI and XbaI.

**pART2-*treC***: *treC_Ec_* was PCR amplified from a genomic DNA preparation of DH5a using primers pART2treC p1 and pART2treC p2. The purified PCR product was digested with BamHI and XbaI and ligated into pART2 digested with the same enzymes.

**pART2-*treF***: *treF_Ec_* was PCR amplified from a genomic DNA preparation of DH5a using primers pART2treF p1 and pART2treF p2. The purified PCR product was digested with BamHI and XbaI and ligated into pART2 digested with the same enzymes.

**pART2-*otsA*-*mCherry*:** *otsA* was PCR amplified from a genomic DNA preparation of *Arthrobacter* strain A3 using primers pART2OM p1 and pART2OM p2. mCherry was PCR amplified from a plasmid pNA585 using primers pART2OM p3 and pART2OM p4. Fusion PCR was used to link *otsA* and mCherry together. The purified PCR product was digested with BamHI and XbaI and ligated into pART2 digested with the same enzymes.

**pCO-*otsA***: P*_otsA_*-*otsA* was PCR amplified from a genomic DNA preparation of *Arthrobacter* strain A3 using primers pCOotsA P1 and pCOotsA P2. The purified PCR product was digested with BamHI and ligated into pSH152 digested with the same enzyme.

**pCO-*otsA_Ec_***: *P_otsA_* was PCR amplified from a genomic DNA preparation of *Arthrobacter* strain A3 using primers pCOotsAEC P1 and pCOotsAEC P2. *otsA_Ec_* was PCR amplified from a genomic DNA preparation of *E. coli* by using primers pCOotsAEC P3 and pCOotsAEC P4. Fusion PCR was employed to link *P_otsA_* and *otsA_Ec_* together. The purified PCR product was digested with BglII and ligated into pSH152 digested with BamHI.

**pCO-*otsB***: *P_otsA_* and *otsB* was PCR amplified from a genomic DNA preparation of Arthrobacter strain A3 using primers pCOotsA P1, pCOotsB P2, pCOotsB P3 and pCOotsB P4. Fusion PCR was used to link *P_otsA_* and *otsB* together. The purified PCR product was digested with BamHI and ligated into pSH152 digested with the same enzymes.

**pCO-*dsbA***: *P_dsbA_-dsbA* was PCR amplified from a genomic DNA preparation of *Arthrobacter* strain A3 using primers pCOdsba P1 and pCOdsba P2. The purified PCR product was digested with BamHI and ligated into pSH152 digested with the same enzymes.

**pCO-*otsA*-*mCherry*:** P*_otsA_*-*otsA*-*mCherry* was PCR amplified from pAIP1 using primers pAIPPotsA p1 and pAIPPotsA p4 and ligated into pSH152 digested with EcoRV.

**pET28-*otsA***：*otsA* was PCR amplified from a genomic DNA preparation of *Arthrobacter* strain A3 using primers 28otsA P1 and 28otsA P2. The purified PCR product was digested with NcoI and HindIII and ligated into pET28a digested with the same enzymes.

**pET28-*otsA_Ec_***: *otsA_Ec_* was PCR amplified from a genomic DNA preparation of DH5α using primers 28otsAEC P1 and 28otsAEC P2. The purified PCR product was digested with NcoI and XhoI and ligated into pET28a digested with the same enzymes.

**pET28-*otsA_ECmu_*:** Two parts of *otsA_ECmu_* were PCR amplified from a genomic DNA preparation of DH5α using primers ECmuP1 with ECmuP2 and ECmuP3 with ECmuP4 respectively. Fusion PCR was used to link the two parts together. The purified PCR product was digested with BspHI and XhoI and ligated into pET28 digested with NcoI and XhoI.

**pET42-*otsA_A3mu_*:** Two parts of *otsA_A3mu_* were PCR amplified from a genomic DNA preparation of DH5α using primers A3muP1 with A3muP2 and A3muP3 with A3muP4 respectively. Fusion PCR was used to link the two parts together. The purified PCR product was digested with NdeI and HindIII and ligated into pET42 digested with the same enzymes.

**List of primers**:

Restriction enzyme recognition sites included in the primer sequence (underlined) are shown in brackets after the primer name. Primers are listed in the 5' to 3' direction.

28otsA P1 (NcoI): TTTCCATGGCACAAACCCGAGTTCTACCC

28otsA P2 (HindIII): TTTAAGCTTTTGTGGTTCGGCCTCCATTGCC

28otsAEC P1 (NcoI): TTTCCATGGGTCGTTTAGTCGTAGTATCTAACC

28otsAEC P2 (XhoI): AAACTCGAGTCGCAAGCTTTGGAAAGGTAGC

p2eotsa p1: TTAGATCTGAGTCGTTTAGTCGTAGTATCTAACC

p2eotsa p2: ATTCTAGACGCAAGCTTTGGAAAGGTAGC

pART2treF p1 (BamHI): TTTGGATCCGCTCAATCAGAAAATTCAAAACC

pART2treF p2 (XbaI): TTTTCTAGAGCCGTACAAACCAATTAAACGG

pART2treC p1 (BamHI): TTTGGATCCGACTAATCTTCCCCACTGGTGG

pART2treC p2 (XbaI): TTTTCTAGACTTCTGTAACCACCAGACAG

Flag otsA p1 (BamHI): AAGGATCCGATGGACTACAAAGACC

Flag otsA P2 (XbaI): TTTCTAGATCATTGTGGTTCGGCCTCC

otsAR36A p2: CCTGTCCACGGGCAGGGCGTTGGAGACAACAATGAAATCG

otsAR36A p3: CGATTTCATTGTTGTCTCCAACGCCCTGCCCGTGGACAGG

pCOotsA P1 (BamHI): TTGGATCCGGCTTGTGCCGTCCGCTCAGC

pCOotsA P2 (BamHI): TTGGATCCTCATTGTGGTTCGGCCTCCATTGCC

pCOotsAEC P1 (BglII): TTAGATCTGGCTTGTGCCGTCCGCTCAGC

pCOotsAEC P2: GGTTAGATACTACGACTAAACGACTCATCTTAGCCTGTGGTGTGG

pCOotsAEC P3: CCACACCACAGGCTAAGATGAGTCGTTTAGTCGTAGTATCTAACC

pCOotsAEC P4 (BglII): TTAGATCTCTACGCAAGCTTTGGAAAGGTAGC

pCOotsB P2: GCCAGAGGTTGCCTCATCTTAGCCTGTGGTGTGG

pCOotsB P3: CCACACCACAGGCTAAGATGAGGCAACCTCTGGC

pCOotsB P4 (BamHI): AAGGATCCTCACGGTGAGATCGCTAAGTTCCC

pCOdsba P1 (BamHI): ATGGATCCTCAAGACAAGTTACTTCTTTGCAGC

pCOdsba P2 (BamHI): ATGGATCCTCCGGCGTCGGAATCTTCTGTGG

pAIPPotsA p1 (BamHI): AAGGATCCCACGGCCGCTACCAACACACC

pAIPPotsA p2: CCTCCTCGCCCTTGCTCACCATTTGTGGTTCGGCCTCCATTGCC

pAIPPotsA p3: GGCAATGGAGGCCGAACCACAAATGGTGAGCAAGGGCGAGGAGG

pAIPPotsA p4(HindIII): ATAAGCTTTTACTTGTACAGCTCGTCCATGCC

A3mup1(NdeI): ACACATATGGCACAAACCCGAGTTCTACCC

A3mup2:CTGGCGGCGTGCTCGTCTGGTGGTGCAATCCGGTTGGAGACAACAATGAAATCGG

A3mup3:TTGCACCACCAGACGAGCACGCCGCCAGTGCCGGTGGCCTCGTCACCGCTCTGGC

A3mup4 (HindIII): CACAAGCTTTTGTGGTTCGGCCTCCATTGCC

Ecmup1(BspHI): GGGTCATGAGTCGTTTAGTCGTAG

Ecmup2:CGGAATCTTCTGTGGAAGTCCTGTCCACGGGCAGGCGGTTAGATACTACGACTAAACGAC

Ecmup3:CCACAGAAGATTCCGACGCCGGATGGCGCCGTTCCCCCGGCGGCCTTGCCGTTGGCATAC

Ecmup4 (XhoI): AACTCGAGCGCAAGCTTTGGAAAGG

**Construction of strains**:

For strains not carrying autonomous plasmids, the integration of suicide vectors by single cross-over homologous recombination was subsequently verified by PCR amplification of integrated sequences. The deleted *otsA* locus of Ar0002 was previously derived by a double cross-over to integrate the ColE1 *ori* region and Km^R^ replacing the *otsA* coding sequence (Chen *et al*, 2011).

**Ar0006:** Strain Ar0006 was obtained by transferring plasmid pART2-otsA-mCherry into strain Ar0001 by electroporation.

**Ar0007**: Strain Ar0007 was generated by integration of plasmid pAIP1 at the *otsA* locus of wild-type strain Ar0001 by a single homologous recombination cross-over.

**Ar0008**: Strain Ar0008 was obtained by transferring plasmid pART2-*treF_Ec_* into strain Ar0001 by electroporation.

**Ar0010**: Strain Ar0010 was obtained by transferring plasmid pART2-*otsA_Ec_* into strain Ar0001 by electroporation.

**Ar0011**: Strain Ar0011 was obtained by transferring plasmid pART2-flag-*otsA*_R36A_ into strain Ar0001 by electroporation.

**Ar0012**: Strain Ar0012 was obtained by transferring plasmid pART2-*treC_Ec_* into strain Ar0001 by electroporation.

**Ar0111**: Strain Ar0111 was generated by integration of plasmid pCO-*otsA* at the ColE1 *ori* locus of the *otsA* deletion mutant strain Ar0002 by single homologous recombination cross-over.

**Ar0112**: Strain Ar0112 was generated by integration of plasmid pCO-*otsA_Ec_* at the ColE1 *ori* locus of the *otsA* deletion mutant strain Ar0002 by single homologous recombination cross-over.

**Ar0113**: Strain Ar0113 was generated by integration of plasmid pCO-*otsB* at the ColE1 *ori* locus of the *otsA* deletion mutant strain Ar0002 by single homologous recombination cross-over.

**Ar0114**: Strain Ar0114 was generated by integration of plasmid pCO-*dsbA* at the ColE1 *ori* locus of the *otsA* deletion mutant strain Ar0002 by single homologous recombination cross-over.

**Ar0115**: Strain Ar0115 was generated by integration of plasmid pCO-*otsA*_R36A_ at the ColE1 *ori* locus of the *otsA* deletion mutant strain Ar0002 by single homologous recombination cross-over.

**Ar0116**: Strain Ar0116 was generated by integration of plasmid pCO-*otsA*-*mCherry* at the ColE1 *ori* locus of the *otsA* deletion mutant strain Ar0002 by single homologous recombination cross-over.

**References**

Ausmees, N., Wahlstedt, H., Bagchi, S., Elliot, M. A., Buttner, M. J., and Flardh, K. (2007). SmeA, a small membrane protein with multiple functions in Streptomyces sporulation including targeting of a SpoIIIE/FtsK-like protein to cell division septa. Mol Microbiol 65, 1458-1473.

Chen, X. M., Jiang, Y., Li, Y. T., Zhang, H. H., Li, J., Chen, X., Zhao, Q., Zhao, J., Si, J., Lin, Z. W.*, et al.* (2011). Regulation of expression of trehalose-6-phosphate synthase during cold shock in *Arthrobacter* strain A3. Extremophiles *15*, 499-508.

Mistry, B. V., Del Sol, R., Wright, C., Findlay, K., and Dyson, P. (2008). FtsW is a dispensable cell division protein required for Z-ring stabilization during sporulation septation in Streptomyces coelicolor. J Bacteriol *190*, 5555-5566.

Sandu, C., Chiribau, C. B., Sachelaru, P., and Brandsch, R. (2005). Plasmids for nicotine-dependent and -independent gene expression in Arthrobacter nicotinovorans and other arthrobacter species. Appl Environ Microbiol *71*, 8920-8924.

Zhang, H., Li, Y., Chen, X., Sheng, H., and An, L. (2011). Optimization of electroporation conditions for Arthrobacter with plasmid PART2. J Microbiol Methods *84*, 114-120.
